# Activation of Fas/FasL pathway and the role of c-FLIP in primary culture of human cholangiocarcinoma cells

**DOI:** 10.1038/s41598-017-14838-3

**Published:** 2017-10-31

**Authors:** Gianluca Carnevale, Guido Carpino, Vincenzo Cardinale, Alessandra Pisciotta, Massimo Riccio, Laura Bertoni, Lara Gibellini, Sara De Biasi, Lorenzo Nevi, Daniele Costantini, Diletta Overi, Andrea Cossarizza, Anto de Pol, Eugenio Gaudio, Domenico Alvaro

**Affiliations:** 1 Department of Surgery, Medicine, Dentistry and Morphological Sciences, University of Modena and Reggio Emilia, Modena, Italy; 2 Department of Movement, Human and Health Sciences, Division of Health Sciences, University of Rome “Foro Italico”, Rome, Italy; 3 Department of Medico-Surgical Sciences and Biotechnologies, Sapienza University of Rome, Rome, Italy; 4 ITAC “Scarabelli-Ghini”, Imola, Italy; 5 Department of Medicine and Medical Specialties, Sapienza University of Rome, Rome, Italy; 6 Department of Anatomical, Histological, Forensic Medicine and Orthopedics Sciences, Sapienza University of Rome, Rome, Italy; 7 Department of Medical and Surgical Sciences for Children and Adults, University of Modena and Reggio Emilia, Modena, Italy

## Abstract

Intrahepatic cholangiocarcinoma (iCCA) represents a heterogeneous group of malignancies emerging from the biliary tree, often in the context of chronic bile ducts inflammation. The immunological features of iCCA cells and their capability to control the lymphocytes response have not yet been investigated. The aims of the present study were to evaluate the interaction between iCCA cells and human peripheral blood mononuclear cells (PBMCs) and the role of Fas/FasL in modulating T-cells and NK-cells response after direct co-culture. iCCA cells express high levels of Fas and FasL that increase after co-culture with PBMCs inducing apoptosis in CD4^+^, CD8^+^ T-cells and in CD56^+^ NK-cells. *In vitro*, c-FLIP is expressed in iCCA cells and the co-culture with PBMCs induces an increase of c-FLIP in both iCCA cells and biliary tree stem cells. This c-FLIP increase does not trigger the caspase cascade, thus hindering apoptotis of iCCA cells which, instead, underwent proliferation. The increased expression of Fas, FasL and c-FLIP is confirmed *in situ*, in human CCA and in primary sclerosing cholangitis. In conclusion our data indicated that iCCA cells have immune-modulatory properties by which they induce apoptosis of T and NK cells, via Fas/FasL pathway, and escape inflammatory response by up-regulating c-FLIP system.

## Introduction

Cholangiocarcinoma (CCA) comprises a heterogeneous group of malignancies emerging at any portion of the biliary tree, representing the second most frequent type of primary liver cancer^1^. On the basis of anatomical location, CCA is currently classified into intrahepatic (i), perihilar (p), and distal (d)^2^. Typical features of iCCAs are the high inter- and intra-tumour heterogeneities and the high representation of cancer stem cell (CSC) and cells expressing epithelial/mesenchymal transition (EMT) traits^3,4^. Moreover, iCCA is characterized by a prominent desmoplastic stroma composed of cancer-associated fibroblasts, inflammatory cells and vascular cells^5,6^.

iCCA arises in the context of bile duct inflammation^7^. From a histological point of view, iCCA could be categorized as a pure mucin adenocarcinoma (mucin-iCCA) or as a mixed-iCCA form with areas of hepatocytic differentiation and of neoplastic ductular proliferation. At a molecular level, integrative molecular analysis identified two different biological subtypes of iCCA, namely the proliferation and inflammation classes^8^. The latter is characterized by the activation of inflammatory pathways and overexpression of different cytokines. Recently, the interactions between tumor macrophages and tumor cells including cancer stem cells (CSCs) have been revealed^6,9^; however, almost nothing is known regarding the interaction between lymphocytes and CCA cells. To this regard, the immunological features of human CCA cells and their capability to control the immune-response have not yet been investigated. We have recently described specific immune-modulatory properties of normal stem cell populations isolated from adult biliary tree that are able to induce a Fas/FasL mediated CD4^+^ and CD8^+^ T-cells apoptosis^10^.

With this background, the aims of the present study were to investigate: (i) the expression of Fas and FasL in primary cultures isolated from human iCCA; (ii) the *in vitro* interactions between CCA cells and human PBMCs and the role of Fas/FasL in inducing T-cells and NK cells apoptosis; (iii) *in situ* the expression of Fas and FasL in human iCCA and their relationship with typical markers of CSC.

## Results

### *In vitro* expression of Fas/FasL in primary cultures of human iCCA

The expression of Fas and FasL was investigated in primary cultures of EpCAM-sorted mucin-iCCA and mixed-iCCA cells by Western Blot (WB) and confocal immunofluorescence analyses.

**Figure 1:**
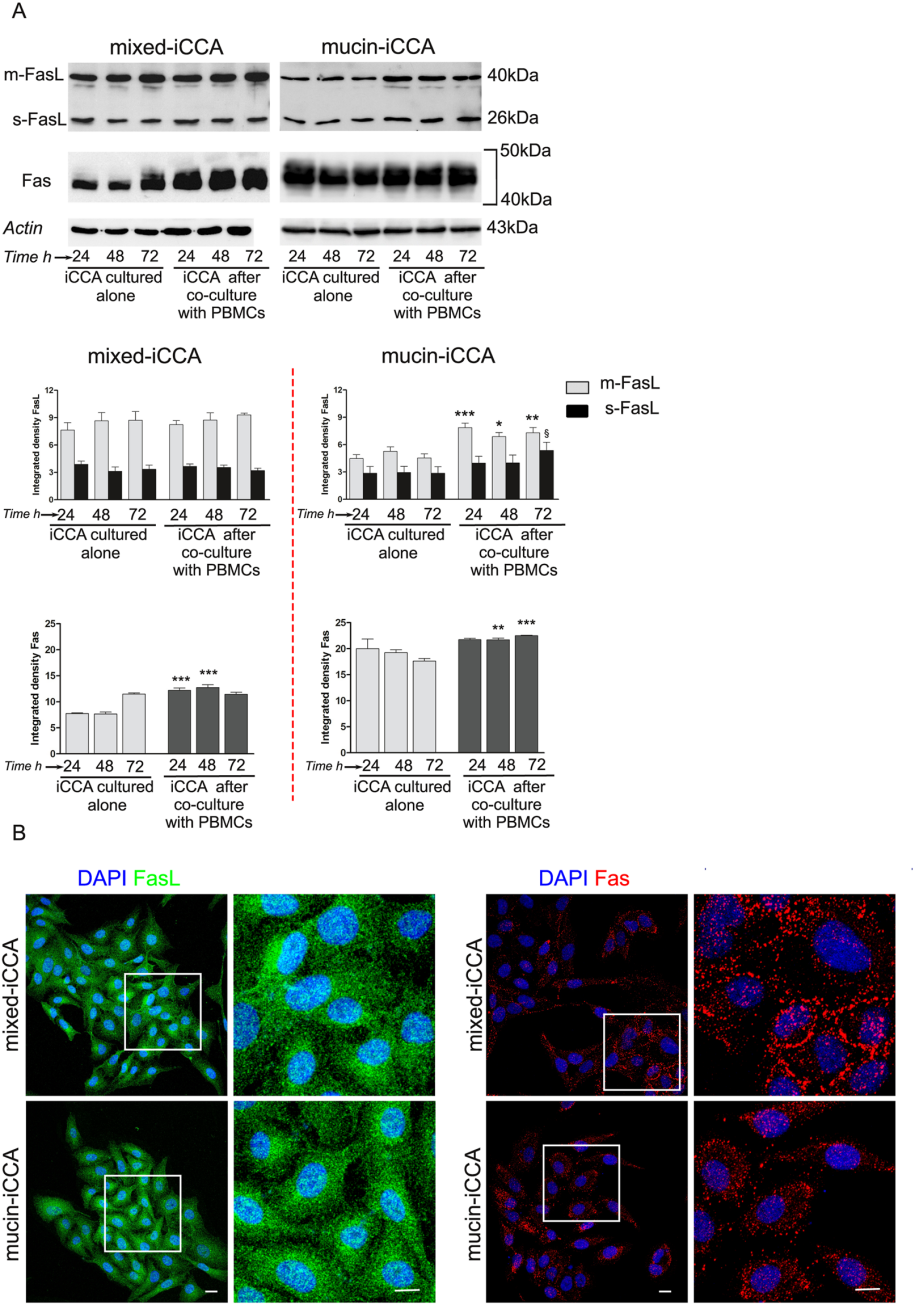
Evaluation of Fas and FasL in intrahepatic cholangiocarcinoma (iCCA) cells. (**A**) Western Blot analysis of Fas and FasL performed on iCCA cells cultured alone and after 24, 48 and 72 h of co-culture with PBMCs; histograms represent mean ± SD; *P < 0.05, **P < 0.01, ***P < 0.001; ^§^P < 0.05 iCCA co-cultured with PBMCs versus iCCA cells cultured alone at the same time points. Statistical analysis was carried out by Student *t-*test. (**B**) Confocal images of iCCA cells labelled by anti-FasL and by anti-Fas Abs. Cells were counterstained by DAPI. The squares in the images indicate magnification area shown on the right side. Scale bar = 10 µm. Full-length blots in (**A**) are presented in Supplementary Figure S2.

WB analysis was performed in both mucin- and mixed-iCCA cells cultured alone and after 24, 48 and 72 h of co-culture with PBMCs. As shown in Fig. 1A, primary cultures of both mixed- and mucin-iCCA subtypes constitutively expressed Fas and FasL. As far as the expression by WB of FasL is concerned, we detected either the membrane form (mFasL), represented by two bands between 37 and 40 kDa, and the soluble form (sFasL), a 26 kDa band. In mixed-iCCA primary cell cultures, a strong expression of both FasL forms was observed in cells cultured alone and in cells maintained from 24 to 72 h in co-culture with PBMCs (Fig. 1A histograms). In contrast, the expression of Fas in mixed-iCCA primary cell cultures was significantly increased after 24 and 48 h of co-culture with PBMCs (*P* < 0.001 vs mixed-iCCA cultured alone at the same time points) (Fig. 1A histograms). Also in mucin-iCCA primary cell cultures, WB analysis demonstrated the expression of membrane and soluble FasL. Interestingly, the expression of membrane form of FasL was significantly increased in mucin-iCCA cells after co-culture with PBMCs (histograms; *P* < 0.001, *P* < 0.01, *P* < 0.05 vs mucin-iCCA cultured alone at the same time points). A significant increase of soluble FasL was also detected in mucin-iCCA cells co-cultured with PBMCs but only after 72 h of culture (histograms; *P* < 0.05). A significant increase of the expression of Fas was also detected in mucin-iCCA cells following co-culture with PBMCs at 48 and 72 h (histograms; *P* < 0.001, *P* < 0.01 vs mucin-iCCA cells cultured alone, Fig. 1A). Cellular localization of FasL and Fas obtained by confocal immunofluorescence analysis was shown in Fig. 1B. According to expectations, a cytoplasmic localization was observed for FasL whereas Fas showed the typical spotted pattern of a membrane receptor.

### Cell proliferation and apoptosis in primary cultures of iCCA co-cultured with PBMCs

**Figure 2:**
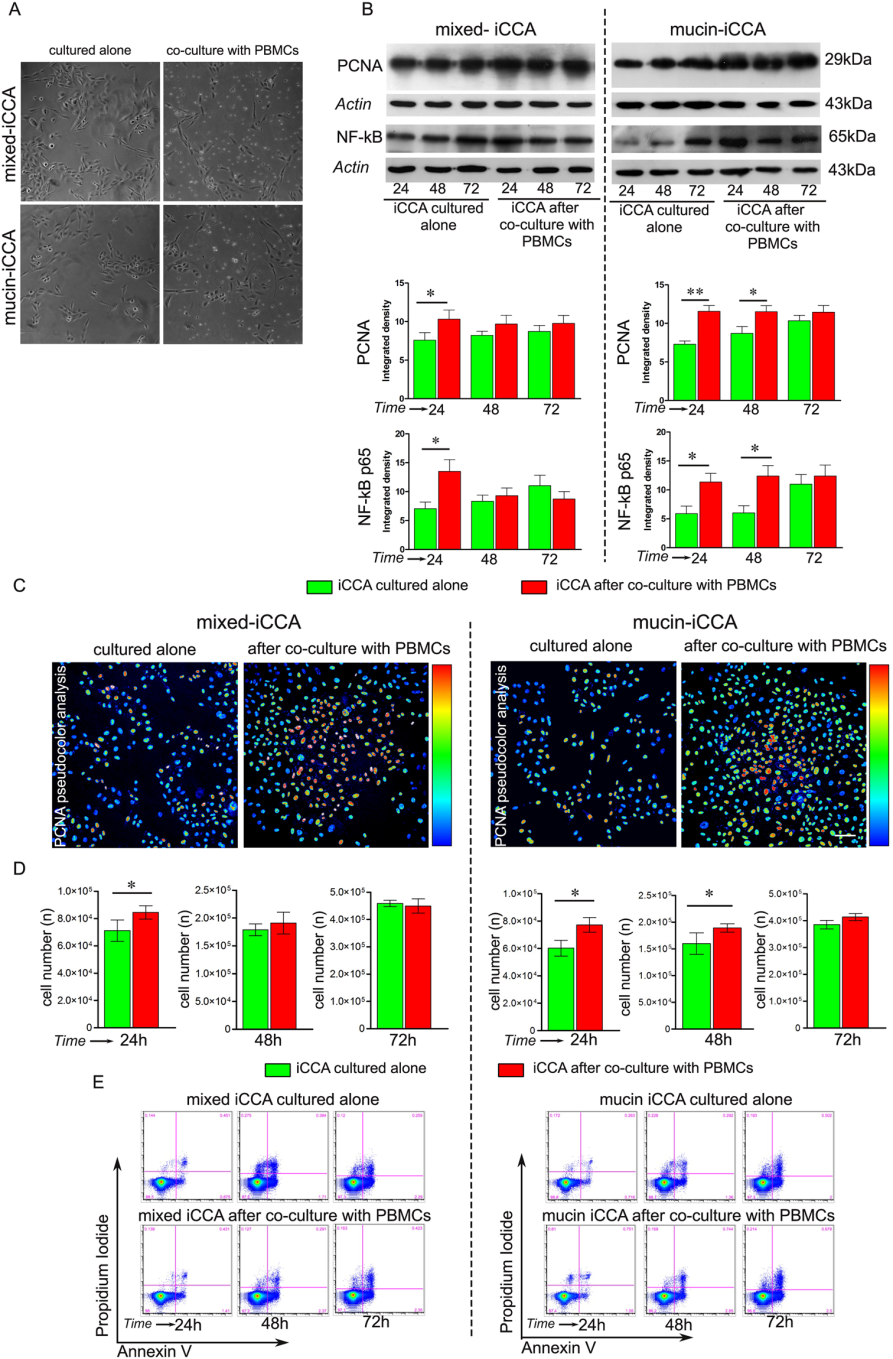
Apoptosis and proliferation in mixed and mucin-iCCA cells. (**A**) Representative phase contrast images of mixed- and mucin-iCCA cells cultured alone and co-cultured with PBMCs. (**B**) Western Blot analysis of PCNA and NF-kB p65 in mixed- and mucin-iCCA cells cultured alone and after co-culture with PBMCs. Actin bands represent a control of protein loading; histograms shown as mean ± SD, **P < 0.01, *P < 0.05, versus iCCA cells cultured alone at the same time points. (**C**) Immunofluorescence and pseudocolor analysis of PCNA immunolabeling in mixed- and mucin-iCCA cells cultured alone and after 24 h of co-culture with PBMCs. Scale bar 50 µm. (**D**) Histograms show the mean ± SD of cancer cell counts obtained from both iCCA cells cultured alone and co-cultured with PBMCs at different time points, *P < 0.05, versus iCCA cells cultured alone at the same time points. (**E**) Representative FACS analysis of Propidium Iodide and Annexin V double stained mixed- and mucin-iCCA cells cultured alone and co-cultured with PBMCs at different time points. Statistical analysis was carried out by Student t-test. Full-length blots in (**B**) are presented in Supplementary Figure S3.

Cell proliferation was evaluated in both mixed- and mucin-iCCA cells, cultured alone and co-cultured with PBMCs (Fig. 2A).

As shown in Fig. 2B, WB analysis of PCNA was carried out in both iCCA subtypes, with densitometry analysis revealing a significant increased expression of PCNA in mixed-iCCA cells after 24 h of co-culture with PBMCs (*P* < 0.05 vs mixed-iCCA cells cultured alone at the same experimental time point, Fig. 2B, histograms). Moreover, WB analysis of NF-kB p65 showed a higher level of expression in mixed-iCCA cells after 24 h of co-culture with PBMCs (*P* < 0.05 vs mixed-iCCA cells cultured alone at the same experimental time point, Fig. 2B, histograms). Similar results were observed in mucin-iCCA cells (Fig. 2B). In particular, densitometry analysis revealed higher levels of expression of PCNA in mucin-iCCA cells after 24 and 48 h of co-culture with PBMCs than in control cells cultured alone (*P* < 0.01, 24 h; *P* < 0.05, 48 h vs mucin-iCCA cells cultured alone at the same time points, Fig. 2B, histograms). Likewise, by WB analysis, NF-kB p65 expression was enhanced in mucin-iCCA cells after 24 and 48 h of co-culture with PBMCs (*P* < 0.05 vs mucin-iCCA cells cultured alone at the same experimental time points).

Data on cell proliferation of both iCCA subtypes were further confirmed by immunofluorescence analysis of PCNA. Indeed, pseudocolor images (Fig. 2C) demonstrated that, after 24 h of exposure to PBMCs, the number of both mixed- and mucin-iCCA cells increased and the level of signal from anti-PCNA antibody showed a higher intensity at nuclear level (Fig. 2C).

Cell counting also demonstrated (Fig. 2D) that cell proliferation rate was increased in mixed-iCCA co-cultured for 24 h with PBMCs (*P* < 0.05 vs mixed-iCCA cells cultured alone, Fig. 2D, histograms) and in mucin-iCCA cells co-cultured for 24 and 48 h with PBMCs (*P* < 0.05 vs mucin-iCCA cells cultured alone at the same experimental time points, Fig. 2D, histograms). As far as apoptosis is concerned, almost all iCCA cells (97–98%) co-cultured with PBMCs were negative for either Propidium Iodide (PI) and Annexin V (Ann-V) labeling at each experimental time point, and no significant differences were observed with respect to iCCA cells cultured alone (Fig. 2E). This demonstrated that no apoptosis induction occurred in mixed and mucin-iCCA cells after PBMCs contact.

Taken together, these findings demonstrated that PBMCs co-cultured with mixed - and mucin-iCCA cells failed to induce apoptosis of cancer cells which, in contrast, showed increased proliferation rates and, likewise, an up-regulation of NF-kB p65.

### FADD, c-FLIP and caspases expression

**Figure 3:**
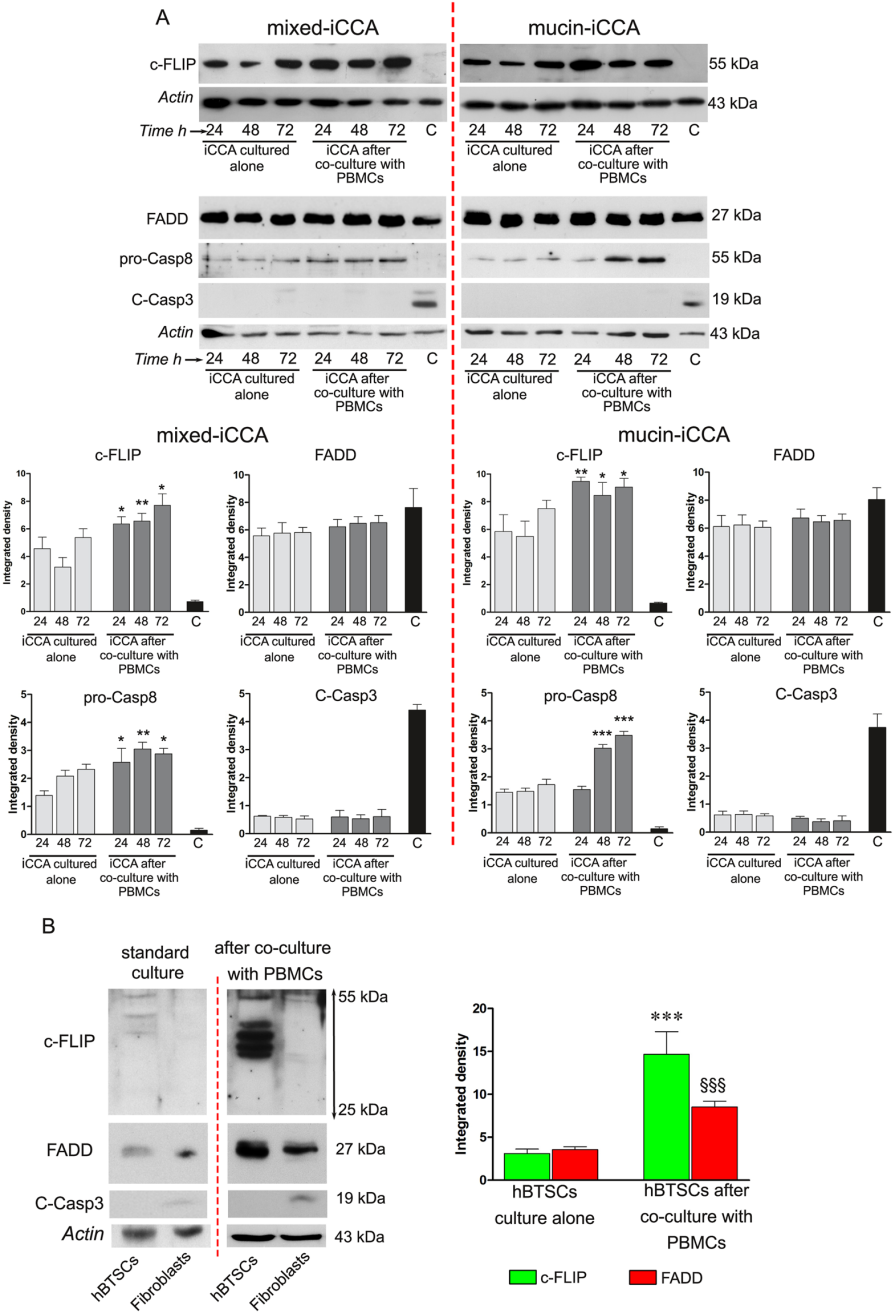
FADD, c-FLIP and caspases expression. (**A**) Western blot analysis of c-FLIP_,_ FADD, pro-caspase 8 and cleaved caspase 3 performed on iCCA cells cultured alone and on iCCA cells after 24, 48 and 72 h of co-culture with PBMCs. Actin bands were presented as control of protein loading; histograms represent mean ± SD, *P < 0.05, **P < 0.01, ***P < 0.001 iCCA cells co-cultured with PBMCs versus iCCA cells cultured alone at the same time points. iCCA cells treated with staurosporine, as indicated with C, were used as control B) Western Blot analysis of c-FLIP, FADD and cleaved caspase 3 performed in hBTSCs and human fibroblasts cultured alone and after 72 h of co-culture with PBMCs. Actin bands were presented as control of protein loading. Histograms represent densitometric analysis of c-FLIP and FADD expression, reported as mean ± SD. ***P < 0.001, ^§§§^P < 0.001, hBTSCs co-cultured with PBMCs vs hBTSCs cultured alone, at 72 h. In any case statistical analysis was carried out by Student *t-*test. Full-length blots in (**A**) and (**B**) are presented in Supplementary Figures S4, S5, S6 and S7, respectively.

FADD, a classical signaling apoptotic adaptor, was evaluated in iCCA cells cultured alone and after co-culture with PBMCs. WB analysis revealed (Fig. 3A) a slight, although not statistically significant, increase in the expression of FADD in both iCCA subtypes after co-culture with PBMCs, in comparison with iCCA cells cultured alone.

Then, we evaluated the expression of the c-FLIP protein, that was constitutively expressed in iCCA primary cultures (Fig. 3A). When iCCA cells were exposed to PBMCs, the expression of c-FLIP significantly increased, without differences between the two iCCA subtypes. In particular, densitometric analysis showed that after 24 h of co-culture with PBMCs both iCCA cells exhibited a strong expression of c-FLIP which was maintained through the whole co-culture time in statistically significant manner (**P < 0.01, *P < 0.05 versus iCCA cells cultured alone at the same experimental time points, Fig. 3A histograms).

This data indicated that the interaction with PBMCs induces a higher expression of c-FLIP. The precursor form of caspase 8 significantly increased when iCCA cells were co-cultured with PBMCs (Fig. 3A histograms; significance was set at *P* < 0.05 vs iCCA cells cultured alone in both iCCA subtypes). Moreover, as shown in Fig. 3A, histograms obtained by densitometric analysis revealed no significant difference with regard to cleaved caspase 3 expression between iCCA cells co-cultured with PBMCs vs iCCA cells cultured alone. These data indicate that the increase of pro-caspase 8 did not reflect the increase of cleaved caspase 3. These data are in accordance with FACS analysis of AnnV/PI labeled cells (Fig. 2E).

As positive controls, primary cell cultures of mixed- and mucin-iCCA cells were treated with 1 µM Staurosporine for 8 h. Densitometric analysis demonstrated that high levels of FADD were still detectable after exposure to staurosporine, whereas c-FLIP was not detected. Moreover, the lack of expression of pro-caspase 8 followed by a clear detection of cleaved caspase 3 demonstrated the effective induction of apoptotic downstream pathway (Fig. 3A).

The expression of FADD, c-FLIP_,_ and cleaved caspase 3 was also evaluated by WB analysis in human biliary tree stem/progenitor cells (hBTSCs; Fig. 3B). hBTSCs expressed low levels of FADD and c-FLIP when cultured alone, whereas the expression of FADD and c-FLIP significantly increased in hBTSCs after 72 h of co-culture with PBMCs (Fig. 3B histograms; *P* < 0.001 vs hBTSCs cultured alone). On the contrary, human fibroblasts, used as positive control, did not express c-FLIP and showed low levels of FADD, that increased, instead, after exposure to PBMCs. At the same time, cleaved caspase 3 was undetectable in hBTSCs alone and after co-culture with PBMCs. In human fibroblasts, data showed an increase of cleaved caspase 3 after exposure to PBMCs (Fig. 3B).

**Figure 4:**
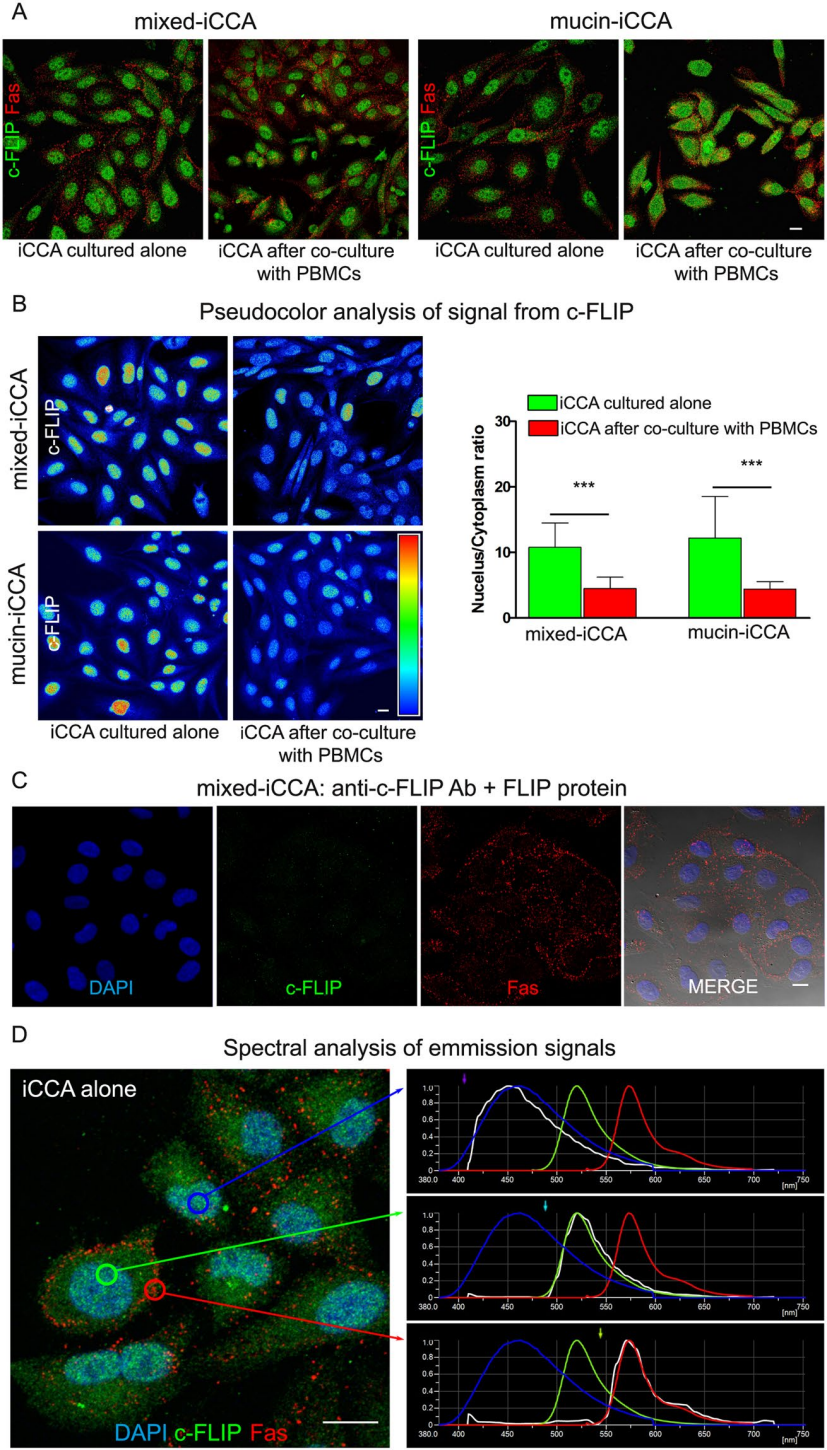
Confocal immunofluorescence analysis of iCCA cells. (**A**) c-FLIP and Fas double labeling of iCCA cells cultured alone and after 24 h of co-culture with PBMCs. Scale bar = 10 µm. (**B**) Pseudocolor analysis of c-FLIP immunolabeling in both iCCA cells. Histograms reported on the right side of pseudocolor images represent the mean ± SD of the ratio of nucleus/cytoplasm expression of c-FLIP in both cancer cell types cultured alone, in comparison with cancer cells co-cultured with PBMCs (***P < 0.001). (**C**) Specificity control of c-FLIP antibody. Sample was counterstained with DAPI and anti-Fas Ab. Fluorescence images were superimposed to DIC image. (**D**) Triple immunofluorescence of mixed-iCCA cells by: DAPI; anti-c-FLIP Ab detected by Alexa488 conjugated secondary Ab; anti-Fas Ab detected by alexa546 conjugated secondary Ab. Spectral confocal analysis of signals from DAPI, alexa488 and alexa546 fluorochromes was shown in the graphs on the right side. Theoretical emission spectra of fluorochromes was represented in the respective color (DAPI in blue; alexa488 in green; alexa546 in red) whereas real emission spectrum was represented in white. Circles in the image indicate areas of analysis.

Confocal immunofluorescence analysis was performed in iCCA primary cell cultures under standard culture conditions and after 24 h of co-culture with PBMCs, in order to evaluate cellular localization of c-FLIP and Fas. As shown in Fig. 4A, the expression of c-FLIP and Fas was detected in cancer cells. In iCCA cells alone, the expression of c-FLIP was mainly localized in the nucleus while, after 24 h of co-culture with PBMCs the expression of c-FLIP decreases in the nucleus and increases in the cytoplasm, as highlighted by pseudocolor images (Fig. 4B). The gray levels analysis of the nucleus/cytoplasm c-FLIP expression ratio showed a statistically significant reduction of the ratio after 24 h of co-culture with PBMCs, with respect to standard culture conditions (n = 5; *P* < 0.001; Fig. 4B). These data suggest that PBMCs induced a reduction in the translocation of c-FLIP from the cytoplasm to the nucleus, in accordance with previous findings^11,12^. In order to exclude artifacts, anti-c-FLIP Ab specificity was tested by incubating iCCA cells with primary Ab pre-incubated with human FLIP protein. The image reported in Fig. 4C shows no signals from c-FLIP, under this condition, suggesting the absence of cross-reactions of the primary Abs. Moreover, spectral analysis carried out in iCCA samples labeled by DAPI, c-FLIP and Fas demonstrated that only specific signals from secondary Abs were detected during confocal acquisition in sequential mode. Indeed, the real emission spectra from the sample (represented in white) matched with the respective theoretical emission spectra of fluorochromes (Fig. 4D).

### Bcl-2 expression

**Figure 5:**
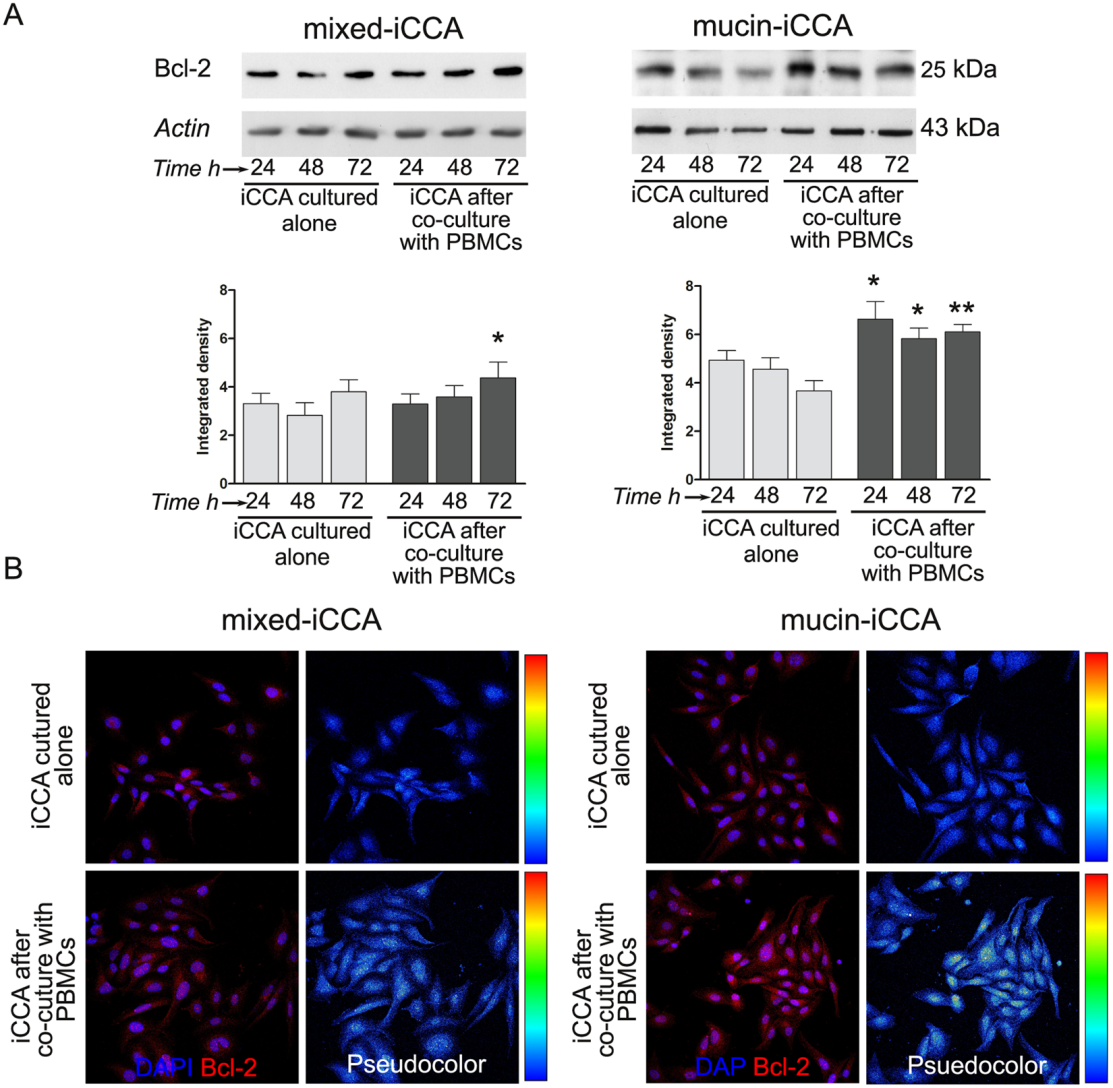
Bcl-2 expression by intrahepatic cholangiocarcinoma (iCCA) cells. (**A**) Western Blot analysis of Bcl-2 performed on iCCA cells cultured alone and co-cultured with PBMCs for 24, 48 and 72 h. Actin bands were presented as control of protein loading. Histograms represent mean ± SD, *P < 0.05, **P < 0.01 iCCA cells co-cultured with PBMCs vs iCCA cells cultured alone at the same time points. Statistical analysis was carried out by Student t-test. (**B**) Confocal analysis and pseudocolor images of Bcl-2 expression in iCCA cells cultured alone and after co-culture with PBMCs at 24 h. Scale bar = 10 µm. Full-length blots in (**A**) are presented in Supplementary Figure S8.

WB analysis (Fig. 5A) showed that Bcl-2 was constitutively expressed under standard culture conditions in iCCA cells. Following exposure to PBMCs, iCCA cells showed a statistically significant increase in the expression of Bcl-2, which occurred in both mixed-iCCA cells (*P* < 0.05 after 72 h of co-culture with PBMCs vs iCCA cells cultured alone at the same time point) and, mostly, in mucin- iCCA cells (*P* < 0.05 after 24 and 48 h, *P* < 0.01 after 72 h of co-culture with PBMCs vs iCCA cells cultured alone at the same time points). The constitutive expression of Bcl-2 and the increase induced by PBMCs exposure (24 h of co-culture) were confirmed by immunofluorescence analysis (Fig. 5B, pseudocolor images next to each immunofluorescence image).

### iCCA cells induce apoptosis of PBMCs

**Figure 6:**
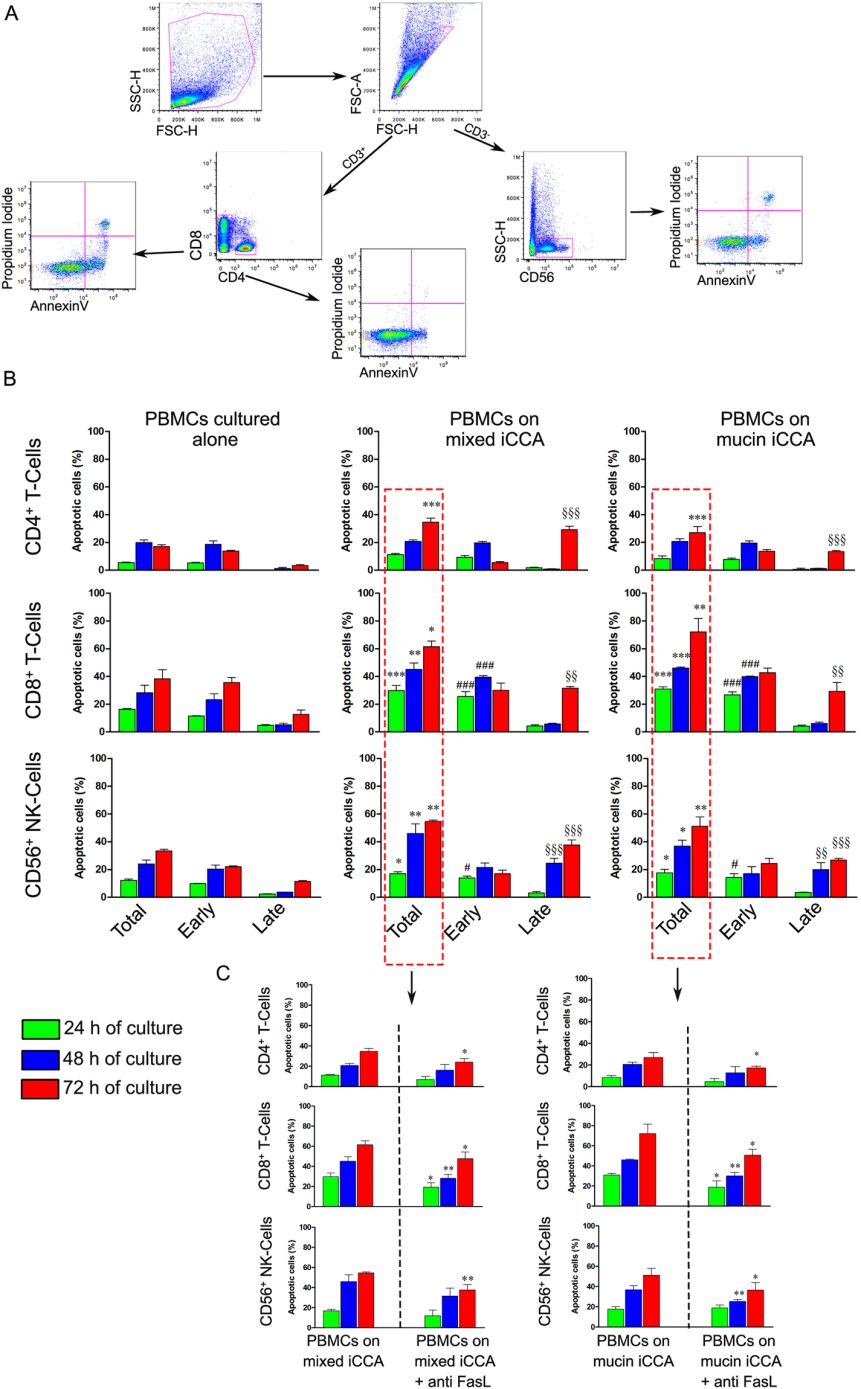
iCCA cells induce apoptosis of PBMCs. (**A**) Gating strategy used to analyze apoptosis of CD4^+^, CD8^+^ T cells and CD56^+^ NK cells. Mononuclear cells were identified by physical parameters (FSC-H vs SSC-H) and cell doublets were removed from the analysis (FSC-H vs FSC-A). CD4^+^, CD8^+^ T cells and CD56^+^ NK cells were identified. In these populations, early apoptotic cells (PI^−^/AnnV^+^) and late apoptotic cells (PI^+^/AnnV^+^, PI^+^/AnnV^−^) were quantified. (**B**) Histograms represent the mean ± SD of the percentage of total apoptotic CD4^+^, CD8^+^ T-cells and CD56^+^ NK cells and their distribution in early apoptosis and late apoptosis, along the whole co-culture time. *P < 0.05, **P < 0.01, ***P < 0.001 total apoptotic CD4+, CD8+ T-cells and CD56+ NK-cells from PBMCs co-cultured with both iCCA cells vs total apoptotic CD4^+^, CD8^+^ T-cells and CD56^+^ NK-cells from PBMCs cultured alone. ^#^P < 0.05, ^###^P < 0.001 early apoptotic CD4^+^, CD8^+^ T-cells and CD56^+^ NK-cells from PBMCs co-cultured with both iCCA cells vs early apoptotic CD4^+^, CD8^+^ T-cells and CD56^+^ NK-cells from PBMCs cultured alone. ^§§^P < 0.01, ^§§§^P < 0.001 late apoptotic CD4^+^, CD8^+^ T-cells and CD56^+^ NK-cells from PBMCs co-cultured with both iCCA cells vs late apoptotic CD4^+^, CD8^+^ T-cells and CD56^+^ NK-cells from PBMCs cultured alone. (**C**) Histograms represent the mean+ SD of the percentage of total apoptotic CD4^+^, CD8^+^ T-cells and CD56^+^ NK-cells from PBMCs co-culture with mixed- or mucin-iCCA and from PBMCs co-culture with mixed- or mucin-iCCA^+^ anti-FasL (*P < 0.05, **P < 0.01 total apoptotic CD4^+^, CD8^+^ T-cells and CD56^+^ NK-cells from PBMCs on mixed and mucin iCCA^+^ anti-FasL vs total apoptotic CD4^+^, CD8^+^ T-cells and CD56^+^ NK-cells from PBMCs on mixed and mucin iCCA).

The cell death occurring in PBMCs co-cultured with cancer cells was evaluated through FACS analysis by examining CD4^+^, CD8^+^ T-cell and CD56^+^ NK cell populations. Figure 6A shows representative plots of gating strategy for flow cytometry analysis performed on PBMCs, to identify CD4^+^, CD8^+^ T-cells and CD56^+^ NK cells. Each sub-population was stained with Annexin V (AnnV) and Propidium iodide (PI) and monitored for 24, 48, and 72 h, in order to obtain AnnV^+^/PI^−^, AnnV^+^/PI^+^ and AnnV^−^/PI^+^. The histograms (Fig. 6B) show the percentage of total apoptotic cells consisting in the sum of early (identified as AnnV^+^/PI^−^ cells) and late apoptotic cells (comprised as AnnV^+^/PI^+^ plus AnnV^−^/PI^+^ cells) and their spitting in early and late apoptosis, along the whole co-culture time.

The total percentage of apoptotic CD4^+^ T-cells obtained from PBMCs co-cultured with iCCA cells increased in a time-dependent manner reaching significant values at 72 h, with respect to CD4^+^ T-cells obtained from PBMCs cultured alone (*P* < 0.001). While no difference was detected in early apoptotic CD4^+^ T-cells, late apoptotic CD4^+^ T-cells significantly increased after 72 h of co-culture (*P* < 0.001, PBMCs co-cultured with iCCA cells vs PBMCs cultured alone) (Fig. 6B).

In CD8^+^ T-cells a significant increase of total apoptotic cells was observed in PBMCs co-cultured with iCCA cells along the whole co-culture time. In particular, analyzing the distribution of apoptotic cells among early stages the most increment was found at 24 and 48 h of co-culture (*P* < 0.001 vs PBMCs cultured alone). Indeed, the significant increase of late apoptotic CD8 cells was observed only after 72 h in co-culture (*P* < 0.01). Similar results were observed for CD56^+^ NK cells. Particularly, FACS analysis revealed a significant increase in the percentage of total apoptotic PBMCs at each time point of co-culture with iCCA cells when compared to PBMCs cultured alone (*P* < 0.05 and *P* < 0.01). Interestingly, the percentage of early apoptotic CD56^+^ NK cells obtained from PBMCs co-cultured with cancer cells increased only at 24 h (*P* < 0.05), while the increase of late apoptotic CD56^+^ NK cells occurred at both 48 and 72 h of co-culture (*P* < 0.01, *P* < 0.001 vs CD56^+^ NK cells obtained from PBMCs cultured alone, Fig. 6B). Experiments of co-culture of PBMCs and fibroblasts were considered, as control, for the evaluation of apoptosis induction in PBMCs (Fig. S1). As shown in Fig. 6C the total percentage of apoptosis in CD4^+^, CD8^+^ T-cell and CD56^+^ NK cell populations was evaluated in PBMCs co-cultured with both iCCA subtypes by adding neutralizing anti-FasL antibody. Interestingly, a significant decrease of total apoptotic CD4^+^ T-cells was observed after 72 h of co-culturing PBMCs with both mixed and mucin iCCA in the presence of anti-FasL (*P* < 0.05 CD4^+^ T-cells from PBMCs on mixed and mucin iCCA + anti FasL vs CD4^+^ T-cells from PBMCs on mixed and mucin iCCA).

By analyzing the total apoptotic CD8^+^ cells, we observed a significant decrease of their percentage as early as at 24 h of co-culture with both iCCA subtypes in the presence of anti-FasL and this was maintained along the whole co-culture time (*P* < 0.05, *P* < 0.01 CD8^+^ T-cells from PBMCs on mixed and mucin iCCA + anti FasL vs CD8^+^ T-cells from PBMCs on mixed and mucin iCCA). As far as CD56^+^ NK cells is concerned, in PBMCs co-cultured with mixed iCCA cells + anti FasL, we observed a significant reduction of total apoptotic cells after 72 h of co-culture (*P* < 0.01 CD56^+^ NK-cells from PBMCs on mixed iCCA + anti FasL vs CD56^+^ NK cells from PBMCs on mixed iCCA). Otherwise, in co-cultures of PBMCs and mucin iCCA cells + anti FasL a significant reduction was already detected after 48 h and was maintained after 72 h (*P* < 0.01, *P* < 0.05 CD56^+^ NK-cells from PBMCs on mucin iCCA + anti FasL vs CD56^+^ NK cells from PBMCs on mucin iCCA).

### *In situ* analyses on normal human liver and human iCCA samples

The expression of FasL and Fas was further confirmed *in situ* on surgical specimens from patients giving informed consent, according to ethical committee statements.

In normal human liver, Fas and FasL were expressed by few cholangiocytes lining interlobular bile ducts (nearly 5–10%; semi-quantitative score: 0.8 ± 0.4). Moreover, the examination of larger intrahepatic bile ducts revealed that nearly 5–10% of PBG cells (semi-quantitative score: 0.7 ± 0.2) showed Fas and FasL labelling.

**Figure 7:**
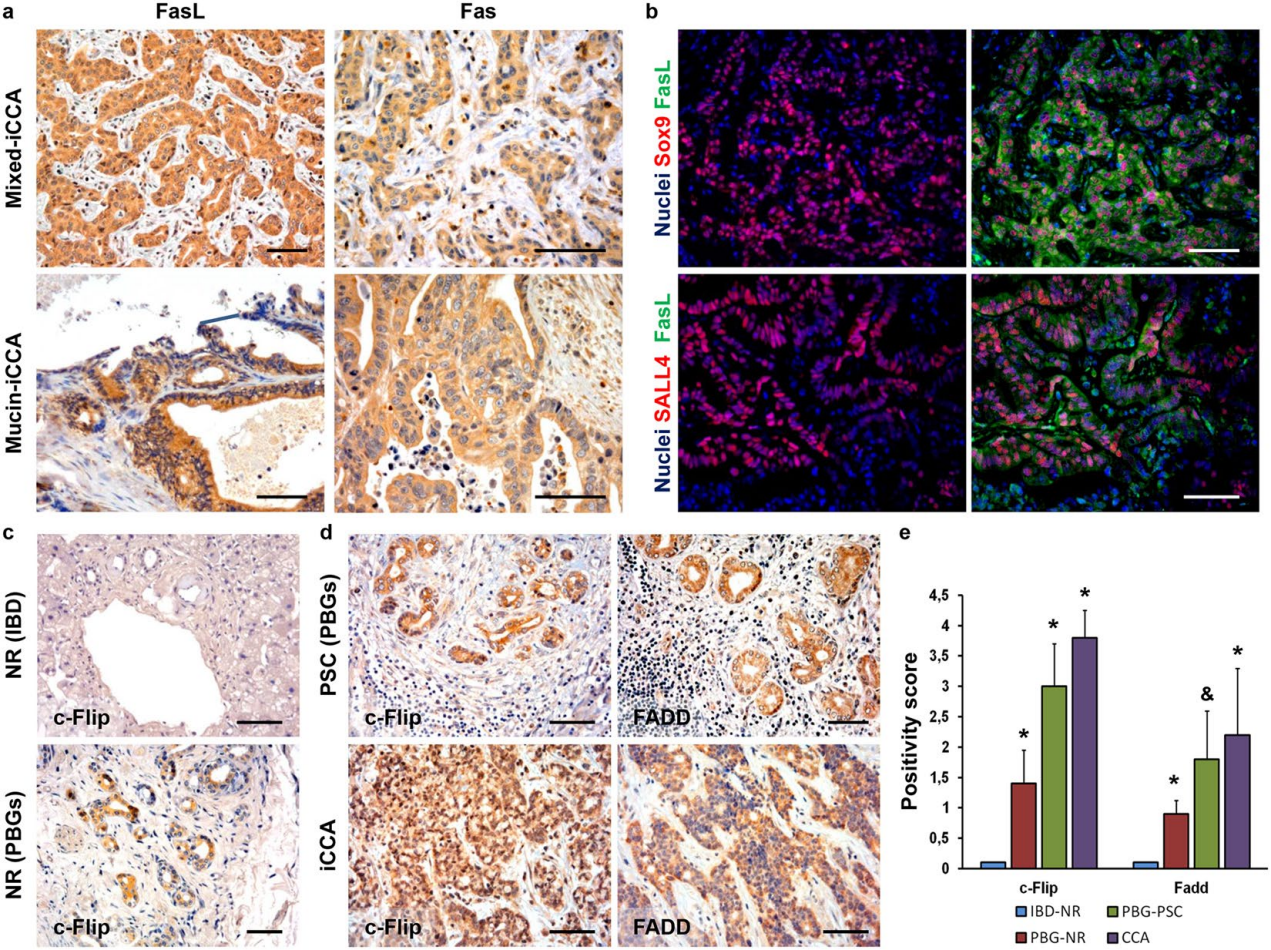
Immunohistochemical study of human intrahepatic cholangiocarcinoma (iCCA) specimens. (**a**) Immunohistochemistry for FasL and Fas in human iCCA samples. FasL and Fas were highly expressed in iCCA. FasL expression resulted higher in small bile duct – type in comparison with large bile duct – type. No differences were found when Fas expression was compared between the two histological sub-types. Original Magnification (O.M.) = 20x. (**b**) Immunofluorescence for FasL and Sox9 or SALL4. FasL resulted co-expressed with SOX9 and SALL4 in the same tumor cells. O.M. = 20x. (**c**,**d**) Immunohistochemistry for c-FLIP and FADD in human samples. In normal (NR) human liver (**c**), cholangiocytes lining normal interlobular bile ducts (IBD) were almost negative for c-FLIP (arrows); differently, cells in peribiliary glands (PBGs) showed high c-FLIP expression (arrowheads). (**d**) c-FLIP and FADD expression in PBG cells was increased in primary sclerosing cholangitis (PSC) and in iCCA samples. O.M. = 20x. Scale bar = 100 µm. (**e**) The histogram shows differences in c-FLIP and FADD expression in different human specimens. *P < 0.05 versus normal samples; ^&^P < 0.01 versus iCCA specimens.

In CCA samples (Fig. 7a), Fas and FasL were highly expressed in iCCA samples (semi-quantitative score: 2.8 ± 0.9) in comparison with cholangiocytes lining interlobular bile ducts and PBG cells examined in normal samples (*P* < 0.05). FasL expression was lower in mucin- (semi-quantitative score: 2.2 ± 0.7) than in mixed-iCCA (semi-quantitative score: 3.5 ± 0.5; *P* < 0.01) while no difference was found as far as Fas expression is concerned (Fig. 7a). Interestingly, Fas-L resulted always co-expressed with the stem cell markers Sox9 and SALL4 (Fig. 7b).

Then, the expression of c-FLIP and FADD was further investigated. In normal human liver, c-FLIP (Fig. 7) was rarely expressed by cholangiocytes lining interlobular bile ducts (less than 5%). Moreover, the examination of larger intrahepatic bile ducts from liver donors revealed that nearly 10–30% of PBG cells showed c-FLIP cytoplasmic and nuclear expression (Fig. 7c). In PSC, a chronic inflammatory disease involving biliary tree and PBGs, an increase of c-FLIP and FADD expression by PBG cells was observed in samples of PSC, when compared with normal PBGs (Fig. 7d; semi-quantitative data are reported in Fig. 7e). In iCCA specimens, the expression of c-FLIP and FADD was markedly higher in comparison with normal cholangiocytes lining interlobular bile ducts and with PBG cells of normal and PSC-affected larger intrahepatic bile ducts (Fig. 7d). No differences were found when c-FLIP and FADD expression were compared between the two histological sub-types of iCCA (mucin versus mixed).

## Discussion

The results obtained in the present study indicated that: i) primary cultures of human mixed- and mucin-iCCA express high levels of FasL and Fas; ii) co-culture with PBMCs induces an increased Fas expression in iCCA cells while FasL increases only in mucin-iCCA cells; iii) in co-culture, PBMCs failed to induce apoptosis of cancer cells and, in contrast, they stimulated tumor cell proliferation and underwent to apoptosis, especially as far as CD4^+^, CD8^+^ T-cells and CD56^+^ NK cells is concerned; iv) FADD and c-FLIP are expressed in hBTSCs and in iCCA cells; v) the co-culture with PBMCs induces: an increase of c-FLIP in both hBTSCs and in iCCA cells, an increase of Bcl-2 in iCCA cells that avoided the activation of downstream apoptotic caspases cascade and, subsequently, the occurrence of intrinsic and extrinsic apoptotic processes; vi) the increased expression of FasL, Fas and c-FLIP observed in samples of human iCCA as well as in PSC, a chronic inflammatory cholangiopathy.

CCA represents a prototype of inflammation-associated cancer since all known risk factors determine a chronic bile duct inflammation including liver flukes and PSC^7,13^. Chronic inflammation can trigger tumor development through different mechanisms^7^. Among inflammatory cells, tumor associated macrophages (TAMs) are the most representative infiltrating immune cells characterizing the CCA microenvironment^6,9^. The high density of TAMs in patients with CCA has been associated with poor prognosis, reduced overall survival and disease-free survival, and metastasis^9^. On the other hand, elevated neutrophil-to-lymphocyte ratio has been shown to be associated with poor anti-tumor immunity and to represent an independent poor prognostic factor in iCCA patients^14^. In parallel, patients affected by iCCA are likely to mount a T-cell immune response against their own tumors and the defects in HLA class I antigen expression in combination with PD-L1 expression by iCCA cells provide them with an immune escape mechanism^15^.

In the present manuscript, we investigated possible cross-talk between PBMCs and tumor cells and mechanisms at the basis of immune escape of cancer cells. Cholangiocarcinoma cells were isolated based on the expression of typical markers, according to previous study demonstrating the expression of CCA stem-like markers^3,9,16^.

Firstly, our results indicated that iCCA cells induce apoptosis in the primary effectors against cancer cells such as T-cells (CD4^+^, CD8^+^) and CD56^+^ NK cells. Second, PBMCs were not able to induce apoptosis in iCCA cells but determined the stimulation of their proliferation. These data are in accordance to previous findings indicating that tumor stem-like cells might modulate their immune-pathological niche according to their necessities, by modelling the macrophage component associated to CSCs^9^.

Then, we aimed to investigate mechanisms at the basis of this immune-escape properties. Lymphocytes express the death ligand FasL and trigger apoptosis through the Fas receptor (CD95) on the target cells^17^. Fas receptor contains an intracellular death domain (DD) which is essential for inducing apoptosis by FasL binding. The DD homotypically attracts the intracellular adaptor protein FADD, which in turn recruits the inactive precursors of caspase 8 and 10. Then, the procaspase 8 and 10 are cleaved and become active initiator caspases. Particularly, active form of caspase 8 may trigger the activation of caspase 3 and/or may induce the intrinsic pathway by cleaving the Bcl-2 family member BID^17^. Moreover, cytotoxic lymphocytes are able to activate directly the apoptotic intrinsic pathway in target cells releasing the cytotoxic secretory granules^18^.

**Figure 8:**
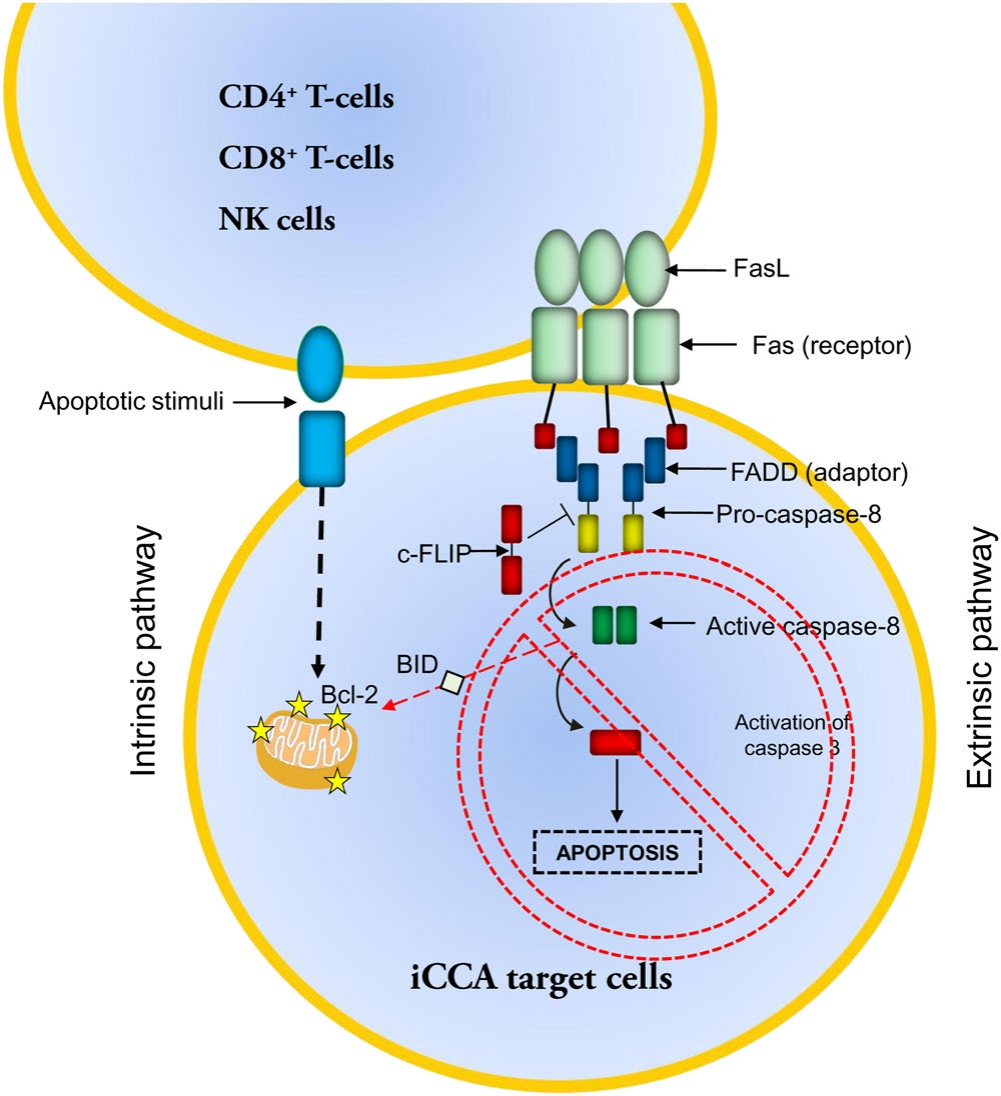
Apoptosis induction through the extrinsic and intrinsic pathways Schematic representation of the extrinsic and intrinsic apoptotic pathways involving FasL; Fas, FADD and c-FLIP.

In general, cancer cells have been shown to be able to escape the apoptotic machinery regulating both extrinsic and intrinsic pathways at different molecular levels^17,19–22^ (Fig. 8.) The present study indicates that CCA cancer cells can modulate apoptotic machinery by the modulation of Fas/FasL pathway and the c-FLIP/FADD cascade. Indeed, iCCA cells over-express both Fas and FasL; the overexpression of CD95 has been correlated with tumor growth promotion, whereas FasL expression could determine apoptosis resistance mechanisms activated by the exposure to inflammatory cells^19,21^. Interestingly, previous *in vivo* observation showed a high level of cell death among lymphocytes infiltrating FasL positive areas of human CCAs^23^. Moreover, our previous report indicated that the activation of Fas/FasL pathway represents a key mechanism by which biliary tree stem/progenitor cells can escape the inflammatory response during their proliferation both *in vitro* and during PSC^10^. In the present manuscript, we further demonstrated that the Fas/FasL pathway is implicated in the immune-modulatory properties of cholangiocarcinoma cells subsets. Particularly, the study of cholangiocarcinoma tissue samples showed that Fas/FasL result co-expressed with stem cell markers in the same tumor cell.

Interestingly, CD95 was shown to be required for the survival of CSC and to allow the emergence of new CSCs^19,20^. In keeping, stimulation of CD95 induced a conversion from non-CSCs to CSCs on multiple tumor cells^19^. This reprogramming activity of CD95 was not due to its apoptotic properties and could represent a mechanism of de-differentiation. Stimulation of CD95 not only increased the number of cancer cells with stem cell traits but also prevented differentiation of CSCs, suggesting that CD95 expression on cancer cells maintains the CSC pool^20^.

*In vitro* study demonstrated that iCCA cells are able to induce apoptosis of CD4^+^, CD8^+^ T-cells and CD56^+^ NK cells and that the rate of apoptosis was reduced by the addition of neutralizing anti-FasL antibody.

Furthermore, the extrinsic pathway may be inhibited directly by procaspase 8 homologue c-FLIP, which forms a heterodymer with the procaspase 8^17–20,22,24^. At the same time, cancer cells may overexpress the anti-apoptotic Bcl-2 proteins thus modulating the intrinsic pathways as well^19,21^. Interestingly, our data were in accordance with this scenario indicating that, when co-cultured with inflammatory cells, iCCA cells increased the expression of c-FLIP and Bcl-2 and this increase is associated with the reduction of apoptosis due to the lack of activation of the caspase cascade. In keeping, c-FLIP/FADD pathway played a role also in the immune-escape of BTSCs. It is noteworthy that in CCA cells c-FLIP and FADD, although being modulated by PBMCs, are constitutively expressed, thus indicating a steadily acquired mechanism to escape apoptosis. On the contrary, the expression of both c-FLIP and FADD in hBTSCs strongly increases only after PBMCs contact, suggesting that an inducible mechanism occurs.

Data collected *in vitro* have been investigated in histological human samples. Our results confirmed that c-FLIP was over-expressed by human iCCA specimens and by hBTSCs in PSC specimens when compared with normal ducts.

From a clinical point of view, our results could have important therapeutic perspectives. CCA represents a high aggressive cancer with poor prognosis and no current curative option^1^. Our data suggest that apoptosis machinery could represent a therapeutic target in iCCA^24^. The use of anti-Bcl-2 mRNA agent (Oblimersen sodium, an 18-base antisense phosphorothioate oligonucleotide) has been studied for the treatment of lymphoma and it has also been tested in combination with other anticancer agents in various cancer types, such as multiple myeloma, small-cell lung cancer, melanoma and non-Hodgkin’s lymphoma^24^. Moreover, downregulation of c-FLIP expression has been induced by combined treatment with carboplatin and thioridazine in human head and neck cancer cells^25^. In keeping, short hairpin RNAs of different c-FLIP isoforms exhibited strong inhibitory effects against the cellular proliferation in melanoma cells^26^. Our results suggest that Bcl-2 and c-FLIP/FADD modulators can be employed in a model to study apoptosis induction strategy in iCCA cells and if this approach could be considered for therapeutic strategy in CCA.

In conclusions, our data indicated that CCA cells have immune-modulatory properties linked to their ability to induce apoptosis in T and NK cells via Fas/FasL pathway and to escape inflammatory response by up-regulating the c-FLIP/FADD system.

## Materials and Methods

### Biliary Tree Stem/Progenitor cell (BTSC) isolation

Normal adult human biliary tissues were isolated from intact livers and pancreata obtained from organ donors at the “Paride Stefanini” Department of General Surgery and Organ Transplantation, Sapienza University of Rome, Rome, Italy. We received the biliary tissues, since most of the biliary tree comprising bile duct, cystic duct, gallbladder and common hepato-pancreatic ampulla, is routinely removed during transplantation procedures. BTSCs were isolated from adult tissues as previously described^10^ and the processing was compliant with Good Manufacturing Practice.

The research protocols were reviewed and approved by the Ethics Committee of Umberto I Policlinico of Rome: Rif 3226/29.05.2014 prot. 609/14 and Rif CE 4492 Prot 295/17. All the experiments were performed in accordance with relevant guidelines and regulation.

### *In situ* immunohistochemistry and immunofluorescence analysis of human CCA samples

Normal specimens were obtained from donors’ livers (n = 5) and extrahepatic biliary tree samples (n = 5) were obtained from organ donors, primary sclerosing cholangitis (PSC) samples (n = 5) were obtained from transplanted livers at the “Paride Stefanini” Department of General Surgery and Organ Transplantation, Sapienza University of Rome, Rome, Italy.

Samples of iCCA (n = 12) and peritumoral noncancerous liver were obtained from patients, namely five women aged 50 to 83 years and seven men, aged 57 to 75 years, presenting a single mass lesion within the liver, which were submitted to curative surgical resection at the “Paride Stefanini” Department of General Surgery and Organ Transplantation, Sapienza University of Rome, Rome, Italy, or at the Surgery, Hepatobiliary Unit, Catholic University of the Sacred Heart School of Medicine, Rome, Italy, or at the Hepato-Biliary Surgery, Regina Elena National Cancer Institute, Rome, Italy. For immunohistochemistry and immunofluorescence analyses, samples were processed as previously described^27–29^. Topographic classification was based on clinical records including surgery reports and pathology diagnosis. No patients underwent to any therapeutic approaches on the tumor before surgery. Detailed protocol is included in the Supplementary Materials and Methods.

### Isolation of iCCA cells

iCCA samples were subjected to mechanical and enzymatic dissociation, as previously described in our laboratories^29,30^. In brief, the tissue was minced with surgical scalpels into fragments of approximately 1 mm^3^. After cutting, the tissue was washed with Dulbeccos’ PBS by bench centrifugation and re-suspended. After washing, fragments were transferred into digestion solution (growth medium with 1 mg/mL collagenase type IV, 0.1 mg/mL hyaluronidase, and 0.1 mg/mL DNase), and incubated for 12 to 16 h at 37 °C in a humidified atmosphere of 5% CO_2_ in air. Then, the cell suspension was filtered with a 100-mm cell strainer placed on a 50-mL tube. The cell strainer was washed with 5 mL of growth medium. The cell suspension was then filtered with a 70-mm cell strainer placed on 50-mL tube. The cell strainer was washed with 5 mL of growth medium.

For magnetic cell sorting, cells were labeled with CD326 MicroBeads (EpCAM, Miltenyi, 130-061-101), and sorted using the Miltenyi Biotec Cell Isolation Kit, according to the manufacturer’s instruction. Isotype-matched mouse immunoglobulins served as controls. Finally, the cells were re-suspended in growth medium and placed into 6-well dish at 37 °C in a humidified atmosphere of 5% CO_2_ in air.

### Isolation of peripheral blood mononuclear cells (PBMCs) and co-culture with iCCA cells

PBMCs were isolated by Ficoll-Hypaque (Life Technologies Italy, Monza) density gradient according to previous report^10^ and re-suspended in RPMI 1640 medium (GIBCO® Life Technologies Italy, Monza) supplemented with 10% FBS, 2 mM glutamine, 100 U/mL penicillin, 100 μg/mL streptomycin (all from Sigma Aldrich, St. Louis, Mo USA).

PBMCs were pre-activated by adding anti-CD3 and the costimulatory anti-CD28 mAbs (1 µg/10^6^ PBMCs, Miltenyi Biotec Inc., Germany; 555330, 555726) to culture medium and then used for co-culture experiments for 24, 48 and 72 h with iCCA cells. Detailed protocol is included in the Supplementary Materials and Methods.

### Flow cytometric analysis

FACS analysis was carried out on both mixed- and mucin-iCCA cultured alone and after co-culture with PBMCs at 24, 48 and 72 h to evaluate whether PBMCs may affect the viability of both iCCA cell types. In parallel, PBMCs cultured alone and PBMCs after 24, 48 and 72 h of co-culture with both iCCA cell types were analyzed by FACS analysis to assess the percentage of apoptosis occurring in CD4^+^ T-cells, CD8^+^ T-cells and CD56^+^ NK cells (all from Biolegend, San Diego, CA; 300526, 301048, 304610). In some experiments, 1 μg/ml of anti-Fas ligand neutralizing antibody (BD, San Jose, California; NOK-1 clone 556372) was added to the co-culture. Detailed protocol is included in the Supplementary Materials and Methods.

### Immunofluorescence and confocal microscopy

The expression of Fas, FasL, c-FLIP and Bcl-2 was evaluated by immunofluorescence confocal analysis in mixed and mucin-iCCA cells cultured alone and after 24 h of co-culture with PBMCs. Samples were processed as previously described^10,31^. Confocal imaging was performed by a Nikon A1 confocal laser scanning microscope as previously described^32^. Particularly, the expression of Fas was evaluated in both living iCCA cell types using a mouse anti-Fas Ab (Cell Signaling Technology, Inc. Danvers, MA USA, 8023) diluted 1:50 in RPMI medium for 15 min. Subsequently, cancer cells were carefully washed with PBS 1X and incubated with secondary Abs diluted 1:100 in RPMI for 15 min and analyzed by live confocal microscopy. For FasL and Bcl-2 analysis, cancer cells were fixed in 4% ice-cold paraformaldehyde for 20 min, permeabilized with 0.1% Triton X-100 in PBS for 5 min, blocked with 3% BSA in PBS for 30 min and then incubated with rabbit anti-FasL (Cell Signaling Technology, Inc. Danvers, MA USA; 4273) and mouse anti-Bcl-2 (DAKO Glostrup Denmark; M0877) abs diluted 1:100 in PBS containing 3% BSA for 1 h at RT. After carefully washing with PBS 1X containing 3% BSA, cells were incubated with the secondary abs diluted 1:200. The double immuno-labeling against c-FLIP and Fas was performed on living cells cultured alone and after 24 h of co-culture with PBMCs. Particularly, cancer cells were incubated for 15 min with mouse anti-Fas (Cell Signaling Technology, Inc. Danvers, MA USA; 8023) Ab diluted 1:50 in RPMI medium, washed with PBS and fixed for 20 min in 4% ice-cold paraformaldehyde in PBS. Samples were then permeabilized with 0.1% Triton X-100 in PBS for 5 min, blocked with 3% BSA in PBS for 30 min and then incubated with rabbit anti-c-FLIP (Santa Cruz Biotechnology, Inc. Texas USA; sc-8347) diluted 1:100 in PBS containing 3% BSA for 1 h at RT. The following secondary Abs were used: donkey anti-rabbit IgG alexa488; donkey anti-mouse IgG alexa488; donkey anti-mouse IgG alexa546 (Life Technologies Corporation, Carlsbad, CA). Nuclei were counterstained by 1 µg/mL 4′,6- diamidino-2-phenylindole (DAPI) in PBS. Negative controls consisted of samples not incubated with the primary Ab. c-FLIP specificity was tested pre-incubating primary Ab with FLIP protein (Novusbio; NBP1-72439) 10 fold more concentrated than primary Ab.

The multilabeling immunofluorescence experiments were carried out avoiding cross-reactions between primary and secondary Abs. Fluorescent samples were observed by a Nikon A1 confocal laser scanning microscope as described below. For multiple detection, the samples were sequentially excited with the respective laser wavelength: 405 nm line of a diode laser for DAPI; 488 nm line of the argon laser for EGFP; 543 nm line of a HeNe laser for Alexa 546. The excitation and the detection of the samples were carried out in sequential mode to avoid overlapping of the two signals. Optical sections were obtained at increments of 0.5 mm in the z-axis and were digitized with a scanning mode format of 1024 × 1024 pixels and 4096 gray levels.

Spectral analysis was carried out to exclude overlapping among multiple signals. In particular samples areas containing two or more fluorochromes was sequentially excited by the respective laser wavelength and the emission signals was superimposed to the theoretical emission spectra of fluorochromes.

The confocal serial sections were processed with ImageJ software to obtain three-dimensional projections and image rendering was performed by Adobe Photoshop Software. Staining intensity of c-FLIP and Bcl-2 expression in both iCCA cell types was evaluated by pseudocolor analysis: blue to white arrays the colors in a spectrum with blue assigned to a lower value than white.

### Western blotting

Whole cell lysates were obtained from mixed- and mucin-iCCA cells cultured alone and co-cultured with PBMCs at different time points, adult hBTSCs and human fibroblasts (cultured alone and after 72 h of co-culture with PBMCs). Furthermore, whole lysates obtained from mixed- and mucin-iCCA cells treated with 1 µM staurosporine for 8 h, were used as apoptosis positive controls. Entire cell lysates were processed as previously described^33^. Detailed protocol is included in the Supplementary Materials and Methods.

### Cell proliferation

The proliferation rate of mixed- and mucin-iCCA cells cultured alone and after co-culture with PBMCs was evaluated at all three experimental time points. Detailed protocol is included in the Supplementary Materials and Methods.

### Statistical analysis

Values are expressed as the mean ± SD obtained by groups of 3–5 samples each. Differences between two experimental samples were analyzed by paired, Student’s t test. Differences between three experimental groups were analyzed by ANOVA followed by Dunnett’s test (GraphPad Prism Software version 5 Inc., San Diego, CA, USA). In any case significance was set at *P* < 0.05.

## Electronic supplementary material

Supplementary data
