# Supplementary material for: Activation of Fas/FasL pathway and the role of c-FLIP in primary culture of human cholangiocarcinoma cells
Source: Sci Rep. 2017 Oct 31;7:14419. doi: 10.1038/s41598-017-14838-3 (PMC5663931; doi:10.1038/s41598-017-14838-3)
Supplement: Supplementary file 1 — Supplementary data [file 41598_2017_14838_MOESM1_ESM.pdf]

***Activation of Fas/FasL pathway and the role of c-FLIP in primary culture of human cholangiocarcinoma cells***

**Authors: Gianluca Carnevale[1], Guido Carpino[2], Vincenzo Cardinale[3], Alessandra Pisciotta[1], Massimo Riccio[1,4], Laura Bertoni[1], Lara Gibellini[1], Sara De Biasi[1], Lorenzo Nevi[5], Daniele Costantini[5], Diletta Overi[6], Andrea Cossarizza[7], Anto de Pol[1], Eugenio Gaudio[6], Domenico Alvaro[5]**

**Affiliations:**

[1]Department of Surgery, Medicine, Dentistry and Morphological Sciences, University of Modena and Reggio Emilia, Modena, Italy. [2]Department of Movement, Human and Health Sciences, Division of Health Sciences, University of Rome "Foro Italico", Rome, Italy. [3]Department of Medico-Surgical Sciences and Biotechnologies, Polo Pontino, [4]ITAC "Scarabelli-Ghini" Imola, Italy, [5]Department of Medicine and Medical Specialties, Sapienza University of Rome, Rome, Italy, [6]Department of Anatomical, Histological, Forensic Medicine and Orthopedics Sciences, Sapienza University of Rome, Rome, Italy. [7]Department of Medical and Surgical Sciences for Children and adults, University of Modena and Reggio Emilia, Modena, Italy,

Table of Contents

**1. Supplementary Materials and methods**
1.1. In situ study of human normal, pathological and CCA samples
1.2. Isolation of peripheral blood mononuclear cells (PBMCs) and co-culture with iCCA cells
1.3. Flow cytometric analysis
14. Western blotting

1.5. Cell proliferation
2. Supplementary References
Figures S1 to S8

## 1. Supplementary Materials and methods

### *1.1. In situ immunohistochemistry and immunofluorescence analysis of human CCA samples*

Specimens were fixed in 10% buffered formalin and embedded in paraffin (55-57°C); serial 3 μm-sections were obtained and stained with Haematoxylin& Eosin, Masson's trichrome, and Periodic Acid Schiff (PAS) staining. For immunohistochemistry, sections were incubated overnight at 4°C with a primary antibody against FasL (rabbit polyclonal; Santa Cruz Biotechnology, code: sc-6237; dilution: 1:50), Fas (mouse monoclonal; Cell Signaling Technology, code: #8023; dilution: 1:100), c-FLIP (rabbit polyclonal; Santa Cruz Biotechnology, code: sc-8347; dilution: 1:50), and FADD (mouse monoclonal; Santa Cruz Biotechnology, code: sc-271520; dilution: 1:50). Samples were rinsed twice with PBS for 5 min, incubated for 20 min at room temperature (RT) with secondary biotinylated antibody, and then with Streptavidin-HRP (LSAB+ System-HRP, Dako, code K0690; Glostrup, Denmark). Diaminobenzidine (Dako) was used as substrate, and sections were counterstained with hematoxylin [S1]. For immunofluorescence, non-specific protein binding was blocked by 5% normal goat serum. Specimens were incubated with primary antibodies against Sox9 (rabbit polyclonal; Millipore, code: AB5809; dilution: 1:50) or SALL4 (rabbit polyclonal; Abcam: Ab29112; dilution: 1:50) and FasL (goat polyclonal; R&D systems: AF126; dilution 1:20). Then, specimens were incubated for 1 hour at RT with labeled isotype-specific secondary antibodies (Alexafluor, Invitrogen, Life Technologies

Ltd, Paisley, UK) and counterstained with DAPI for visualization of cell nuclei. For all immunoreactions, negative controls were also included [S2].

Sections were examined by Leica Microsystems DM 4500 B Microscope (Weltzlar, Germany) equipped with a Jenoptik Prog Res C10 Plus Videocam (Jena, Germany). Immunohistochemistry and Immunofluorescence observations were processed with an Image Analysis System (IAS - Delta Sistem) and were independently performed by two researchers in double blind fashion.

On the basis of histo-morphological aspect and immunohistochemical profile, iCCAs were classified as mucin (large bile duct type) and mixed (small bile duct type) subtypes, as elsewhere [S3].

For quantitative analysis, slides were scanned by a digital scanner (Aperio CS2 for bright field and Aperio FL for immunofluorescence, Aperio Technologies, Inc, Oxford, UK) and processed by ImageScope. The number of positive cells has been calculated as the percentage of positive cells with respect to the total number of cells within tumor mass. According to the percentage of positive cells, data were further expressed using a semi-quantitative scoring systems as following: − (0) = less than 5% positive cells; + (1) = 5–10%; ++ (2) = 11–30%; +++ (3) = 31–50%; ++++ (4) = >50% (14).

*1.2. Isolation of peripheral blood mononuclear cells (PBMCs) and co-culture with iCCA cells*

PBMCs were isolated by Ficoll-Hypaque (Life Technologies Italy, Monza) density gradient according to previous report [S4] and re-suspended in RPMI 1640 medium (GIBCO® Life Technologies Italy, Monza) supplemented with 10% FBS, 2 mM glutamine, 100 U/mL penicillin, 100 µg/mL streptomycin (all from Sigma Aldrich, St. Louis, Mo USA).

PBMCs were pre-activated by adding anti-CD3 and the costimulatory anti-CD28 mAbs (1µg/$10^6$ PBMCs, BD Pharmigen, code: 555330, 555726) to culture medium and then

used for co-culture experiments [S4]. For co-culture experiments, mixed- and mucin-iCCA were cultured upon reaching 70% confluence in expansion medium [αMEM/RPMI (GIBCO® Life Technologies Italy, Monza) plus 10 % FBS, 2 mM L-glutamine, 100 U/mL penicillin, 100 μg/mL streptomycin (all from Sigma Aldrich, St. Louis, Mo USA)], at 37°C and 5% CO2. 24 h prior to the co-culture experiments both iCCA cell types were harvested and seeded at the density of 2000 cells/cm2 in RPMI 1640 (GIBCO® Life Technologies Italy, Monza) medium supplemented with 10 % FBS, 2 mM L-glutamine, 100 U/mL penicillin, 100 μg/mL streptomycin (all from Sigma Aldrich, St. Louis, Mo USA). Co-cultures were obtained by adding pre-activated PBMCs suspension to both iCCA cell types in 6 or 12 well culture plates, at a 10:1 ratio with respect to iCCA cells (Corning, NY USA) or in 2-well Chambered Coverglass (Nalge-Nunc International, NY USA). Co-cultured cells and controls consisting in iCCA cells cultured alone and pre-activated PBMCs cultured alone, were maintained in culture for 24, 48 and 72 hours respectively. Each experiment was performed in triplicate.

*1.3. Flow cytometric analysis*

FACS analysis was carried out on both mixed- and mucin-iCCA cultured alone and after co-culture with PBMCs at 24,48 and 72 hours to evaluate whether PBMCs may affect the viability of both iCCA cell types. In parallel, PBMCs cultured alone and PBMCs after 24, 48 and 72 hours of co-culture with both iCCA cell types were analyzed by FACS analysis to assess the percentage of apoptosis occurring in $CD4^+$ T-cells, $CD8^+$ T-cells and $CD56^+$ NK cells. The gating strategy for flow cytometry analysis performed on PBMCs allowed to identify $CD4^+$, $CD8^+$ T-cells and $CD56^+$ NK cells; cells were identified by physical parameters (FSC-H vs SSC-H) and doublets were removed from the analysis (FSC-H vs FSC-A). Cells were gated on $CD3^+$ T cells and, among these, $CD4^+$ and $CD8^+$ T cells were identified. Among $CD3^-$ T cells, NK cells were identified based on the expression of CD56.

.

Particularly, PBMCs were stained with fluorochrome-labeled mAbs at previously defined optimal concentrations. The following mAbs were used: anti-CD3 PB, anti-CD4 AF700, anti-CD8 BV510, CD56 APC (all from Biolegend, San Diego, CA, code 300417, 300526, 301048, 304610). Cancer cells and PBMCs were incubated for 20 min at room temperature, washed with Stain Buffer (Becton Dickinson, DB, San José, CA) and then re-suspended in Annexin binding Buffer. Cells were stained and incubated with Annexin V Alexa Fluor 488 and 10mg/ml Propidium Iodide according to standard procedure (all from ThermoFisher, Eugene, OR, code A13201, P3566). Cells were immediately acquired on an acoustic focusing flow cytometer (Attune NxT, ThermoFisher). Data were acquired in list mode using Attune NxT 2.4 software and analyzed by FlowJo 9.9 (Tree Star Inc., Ashland, Oregon, USA). Samples were compensated by software after acquisition. Single staining and Fluorescence Minus One controls were performed for all mAbs of the panel to set proper compensation and define positive signals. Apoptosis data are expressed as percentage of total apoptosis (consisting in the sum of early and late apoptosis) and their distribution among early apoptosis ($PI^-$/AnnexV$^+$ cells) and late apoptosis ($PI^+$/AnnexV$^+$plus $PI^+$/AnnexV$^-$ cells). In some experiments, 1 µg/ml of anti-Fas ligand neutralizing antibody (NOK-1 clone; BD, San Jose, California; code 556372) were added to the co-cultureIn any case the experiments were performed in triplicate.

*1.4. Western blotting*

Cell lysates were obtained from mixed- and mucin-iCCA cells cultured alone and after co-cultured with PBMCs at different time points, adult hBTSCs and human fibroblasts (cultured alone and after 72 h of co-culture with PBMCs). Furthermore, whole lysates obtained from mixed- and mucin-iCCA cells treated with 1µM staurosporine for 8 hours, were used as apoptosis positive controls. Cell lysates were processed as previously described [S5]. Whole cell lysates were obtained by using an hypotonic buffer (30 mM

Tris-Cl, pH 7.8, containing 1% Nonidet P40, 1 mM EDTA, 1 mM EGTA, 1 mM $Na_3VO_4$, and freshly added Sigma-Aldrich Protease Inhibitor Cocktail). Lysates were cleared by centrifugation and the lysate was immediately boiled in SDS sample Buffer (62.5mM Tris-HCl pH 6.8; 2% SDS ; 10% glycérol; 100 mM DTT; 5% 2-Mercaptoethanol 0.01mg/ml bromphenol blue). 30 µg of total proteins from each sample were separated by SDS-PAGE and then transferred to PVDF membranes. Blots were incubated with primary Abs: rabbit anti-FasL, mouse anti-Fas, rabbit anti-cleaved Caspase 3 (all from Cell Signaling Technology, Inc. Danvers, MA USA, code); rabbit anti- c-FLIP (R&D system AF821), mouse anti-FADD, mouse anti-pro-caspase 8, (Santa Cruz Biotechnology, Inc. Texas USA, code sc-271520, sc-56070); mouse anti-Bcl-2 (DAKO Glostrup Denmark, code M0877). Primary Abs were diluted 1:1000 and revealed by HRP conjugated secondary Abs (anti-rabbit and anti-mouse, ThermoFisher Scientific, Waltham, MA USA) diluted 1:3000. All membranes were revealed by using Enhanced Chemio Luminescence (Amersham, UK). Anti-actin Ab (Santa Cruz Biotechnology, Inc. Texas USA, code sc-1616) was used as control of protein loading. Densitometry was performed on three independent experiments by ImageJ software. An equal area was selected inside each band and the integrated density was calculated. Data were then normalized to values of background and of control actin band and the values were expressed as mean ± SD.

*1.5. Cell proliferation*

In order to evaluate the proliferation rate of mixed- and mucin-iCCA cells cultured alone and after co-culture with PBMCs at all three experimental time points, cells were plated at the density of 2000 cells/$cm^2$ [αMEM/RPMI (GIBCO® Life Technologies Italy, Monza) plus 10% FBS, 2 mM L-glutamine, 100 U/mL penicillin, 100 µg/mL streptomycin (all from Sigma Aldrich, St. Louis, Mo USA)], at 37°C and 5% $CO_2$. Upon reaching cell adhesion, medium was replaced with RPMI plus 10 % FBS, 2 mM L-glutamine, 100 U/mL penicillin,

100 μg/mL streptomycin and PBMCs were added in each well containing adherent iCCA cells at the ratio of 10:1 with respect to iCCA cell seeding. After 24, 48 and 72 hours of co-culture, PBMCs were removed and after careful washing, adherent iCCA cell types were trypsinized, re-suspended and counted. Mixed- and mucin-iCCA cells cultured alone were used as control. The number of cells was expressed as mean ± SD. Cell proliferation was also evaluated by WB analysis and immunofluorescence analysis of PCNA and NF-kB p65 expression by using the specific primary antibodies mouse anti-PCNA (Millipore clone PC10, MABE288) and mouse anti-NF-kB p65 (Santa Cruz Biotechnology, sc-109). Each experiment was performed in triplicate.

**2. Supplementary References**


S1. Onori P, Alvaro D, Floreani AR, Mancino MG, Franchitto A, Guido M, et al. Activation of the IGF1 system characterizes cholangiocyte survival during progression of primary biliary cirrhosis. J Histochem Cytochem 2007;55:327-334.

S2. Carpino G, Renzi A, Cardinale V, Franchitto A, Onori P, Overi D, et al. Progenitor cell niches in the human pancreatic duct system and associated pancreatic duct glands: an anatomical and immunophenotyping study. J Anat 2016;228:474-486.

S3. Komuta M, Govaere O, Vandecaveye V, Akiba J, Van Steenbergen W, Verslype C, et al. Histological diversity in cholangiocellular carcinoma reflects the different cholangiocyte phenotypes. Hepatology 2012;55:1876-1888

S4. Riccio M, Carnevale G, Cardinale V, Gibellini L, De Biasi S, Pisciotta A, et al. Fas/Fas ligand apoptosis pathway underlies immunomodulatory properties of Human Biliary Tree Stem/Progenitor Cells. J Hepatol 2014;61:1097-1105

S5. Carnevale G, Pisciotta A, Riccio M, De Biasi S, Gibellini L, Ferrari A, et al. Optimized Cryopreservation and Banking of Human Bone-Marrow Fragments and Stem Cells. Biopreserv Biobank 2016;14:138-148

Figure S1

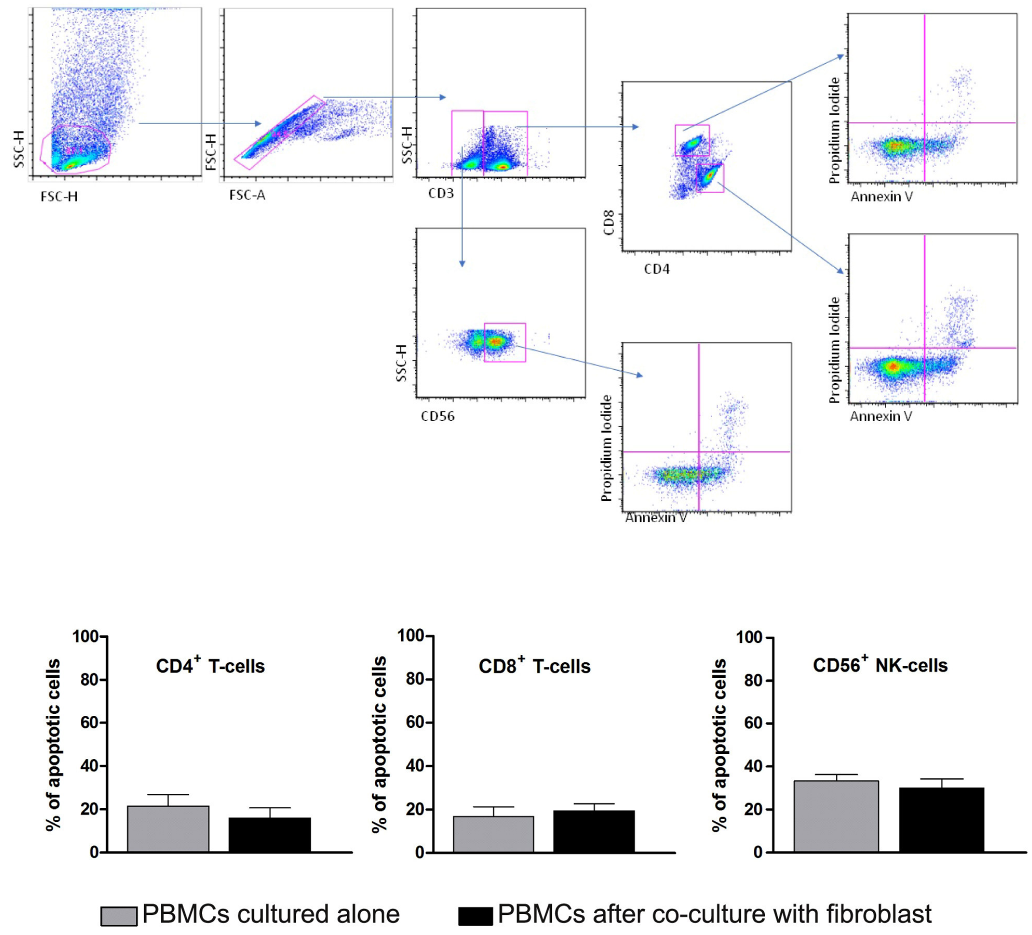


Figure S1: Gating strategy used to analyze apoptosis among CD4+, CD8+ T cells and CD56+ NK cells after 72 hours of co-culture. Mononuclear cells were identified by physical parameters (FSC-H vs SSC-H) and cell doublets were removed from the analysis (FSC-H vs FSC-A). Cells were gated on $CD3^+$ T cells and, among these, $CD4^+$ and $CD8^+$ T cells were identified. Among $CD3^-$ T cells, NK cells were identified based on the expression of CD56. Histograms represent the mean± SD of the percentage of total apoptotic (consisting in the sum of early and late apoptotic cells) $CD4^+$, $CD8^+$ T-cells and $CD56^+$ NK cells obtained from PBMCs cultured alone and from PBMCs co-cultured with fibroblast after 72 hours of direct contact. No statistically significant differences were observed between the two groups.

Figure S2

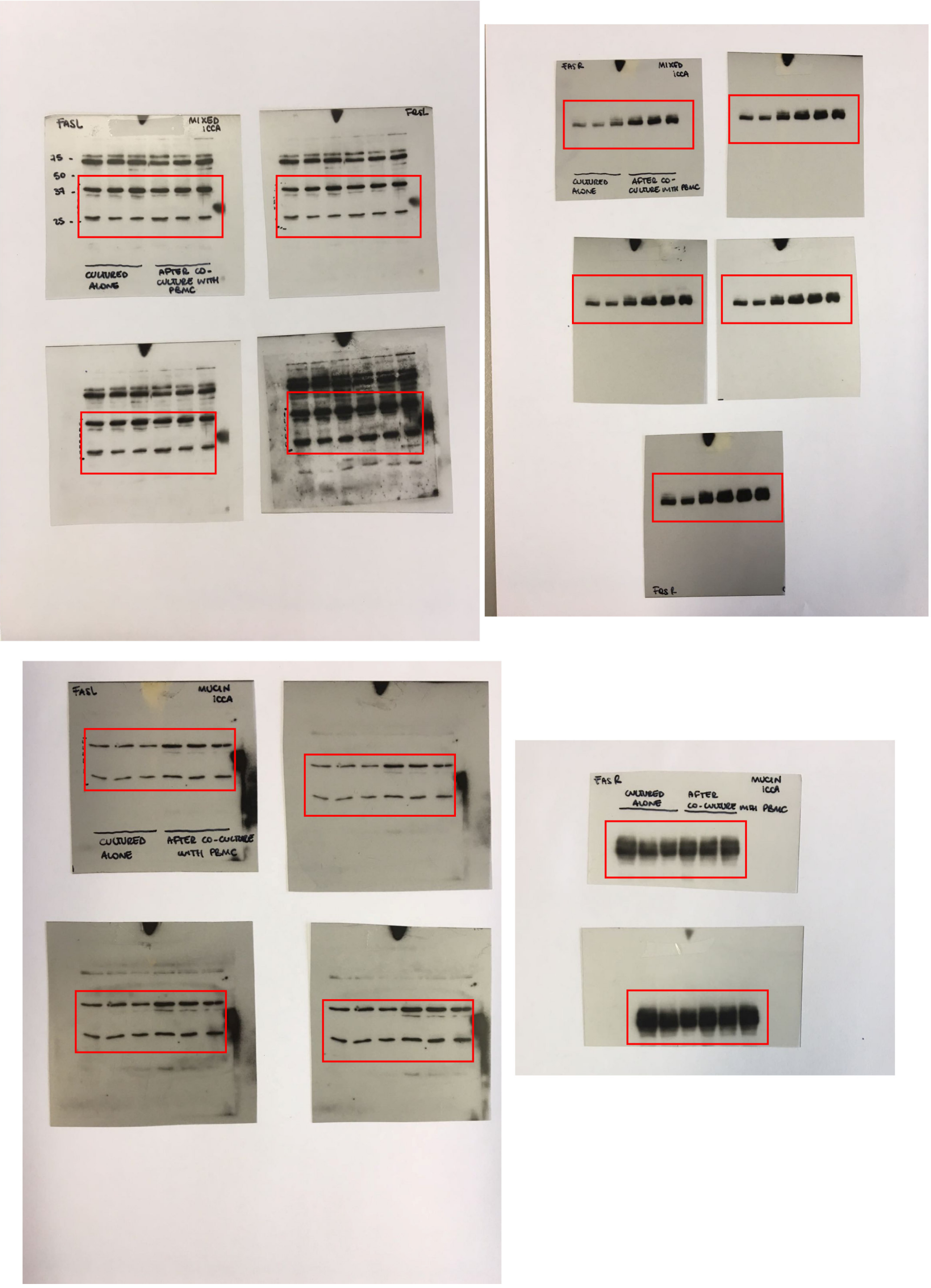


Figure S2. Full-length blots at different exposure times used to generate Figure 1A .

Figure S3

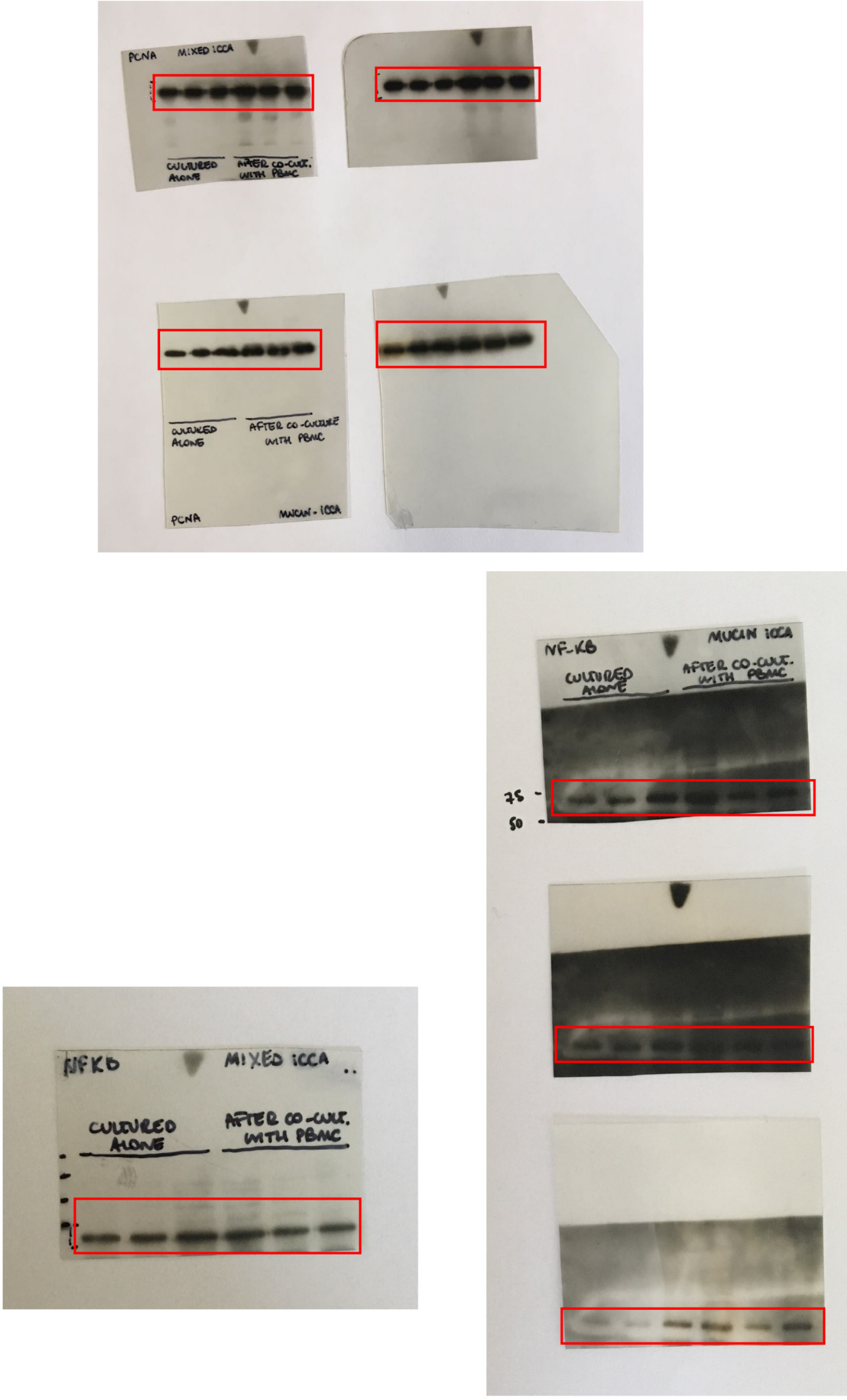


Figure S3. Full-length blots at different exposure times used to generate Figure 2B.

Figure S4

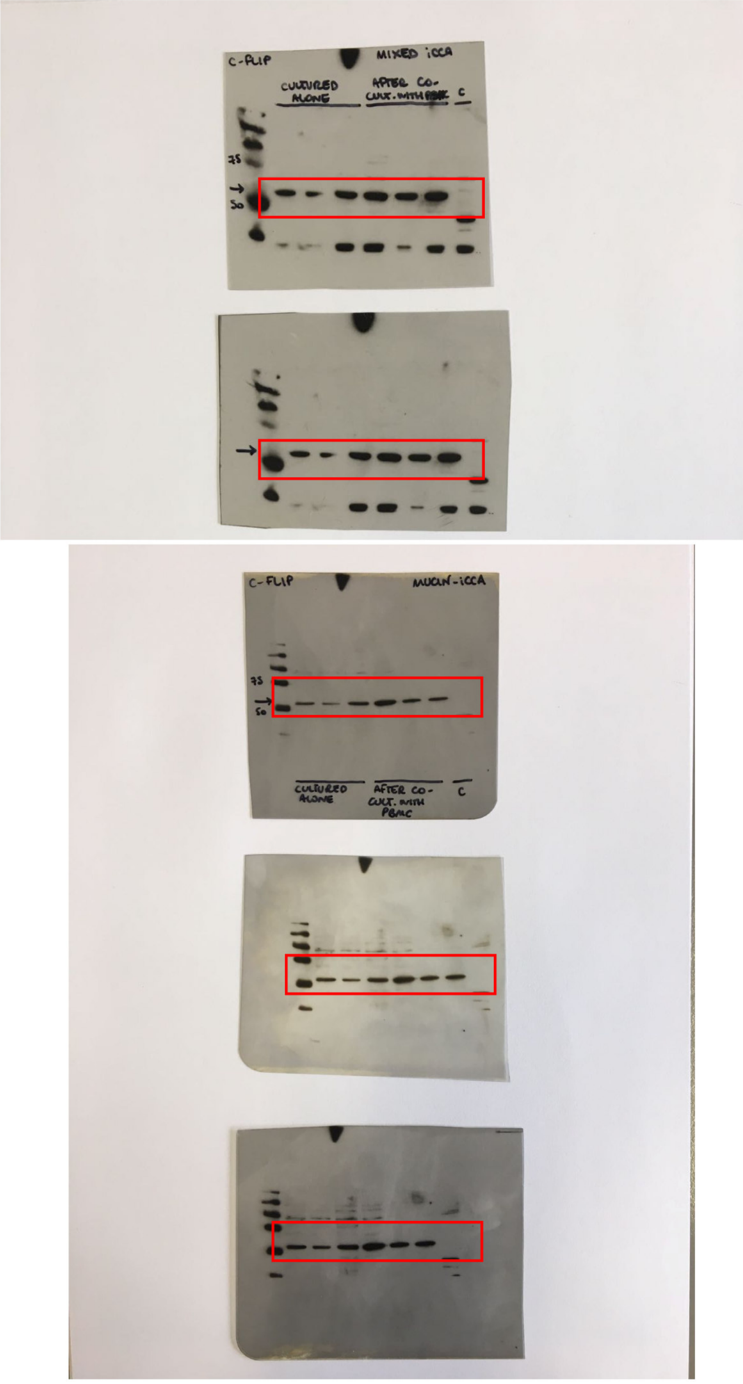


Figure S4. Full-length blots at different exposure times used to generate Figure 3A.

Figure S5

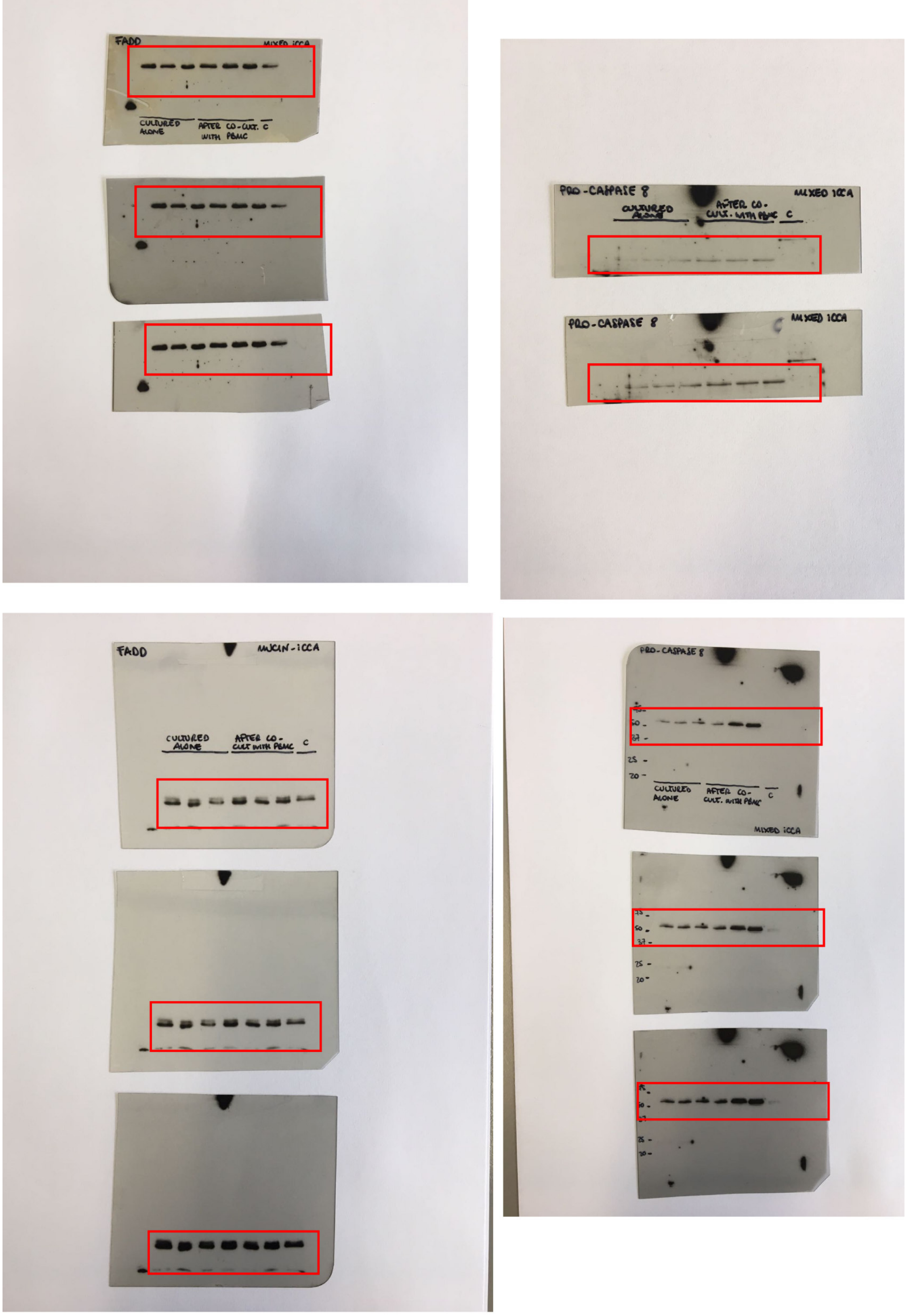


Figure S5. Full-length blots at different exposure times used to generate Figure 3A.

Figure S6

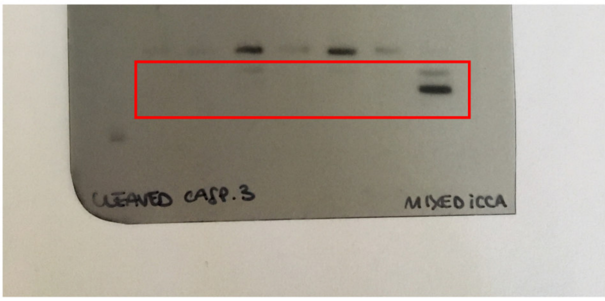


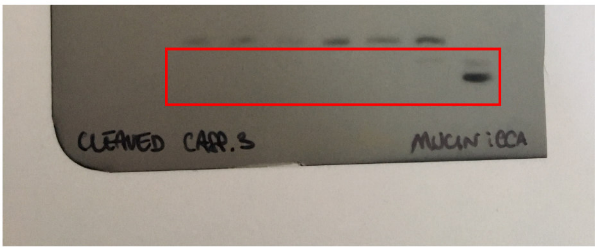


Figure S6. Full-length blots used to generate Figure 3A.

Figure S7

standard culture

c-FLIP FADD cleaved caspase 3

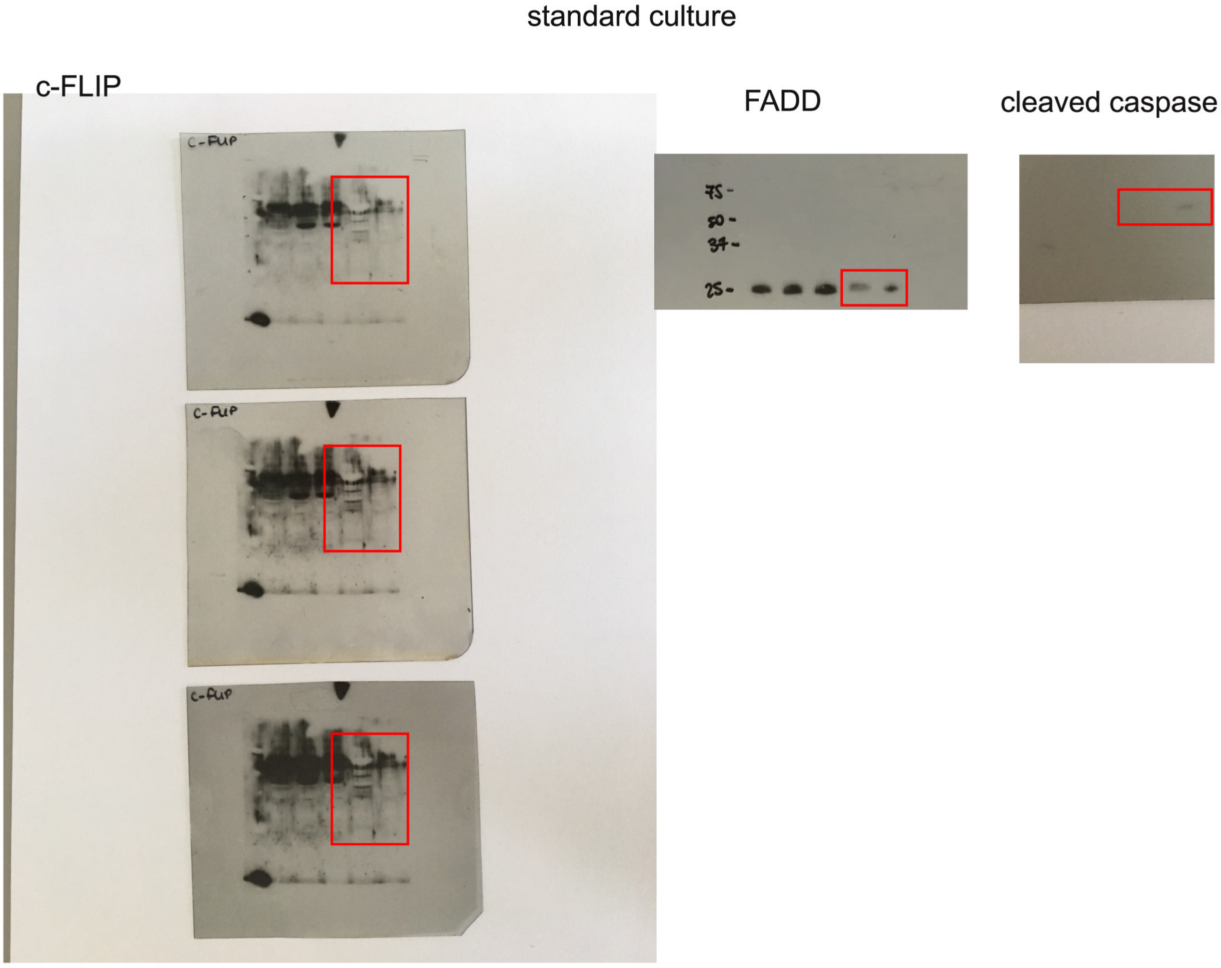

after co-culture

c-FLIP FADD cleaved caspase 3

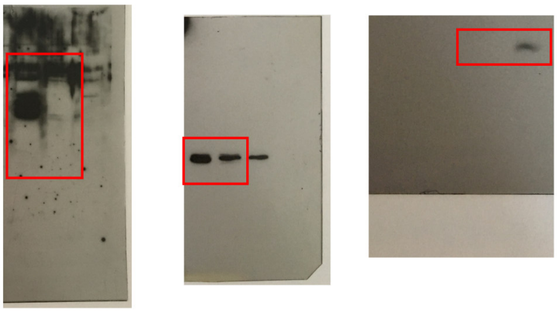

Figure S7. Full-length blots at different exposure times used to generate Figure 3B.

Figure S8

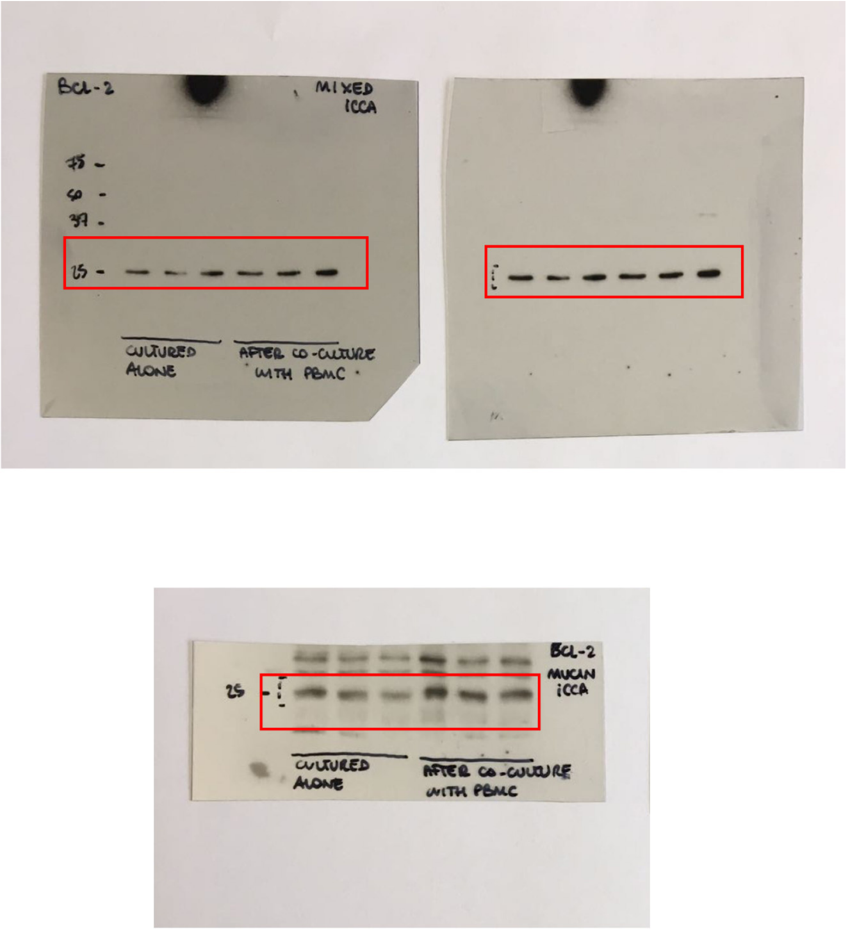


Figure S8. Full-length blots at different exposure times used to generate Figure 5A.
